# Recent Outbreaks of Shigellosis in California Caused by Two Distinct Populations of *Shigella sonnei* with either Increased Virulence or Fluoroquinolone Resistance

**DOI:** 10.1128/mSphere.00344-16

**Published:** 2016-12-21

**Authors:** Varvara K. Kozyreva, Guillaume Jospin, Alexander L. Greninger, James P. Watt, Jonathan A. Eisen, Vishnu Chaturvedi

**Affiliations:** a Microbial Diseases Laboratory, California Department of Public Health, Richmond, California, USA; b Genome Center, Department of Evolution and Ecology, Department of Medical Microbiology and Immunology, University of California, Davis, Davis, California, USA; c Division of Communicable Disease Control, California Department of Public Health, Richmond, California, USA; Swiss Federal Institute of Technology Lausanne

**Keywords:** California, *Shigella sonnei*, shigellosis

## Abstract

*Shigella sonnei* has caused unusually large outbreaks of shigellosis in California in 2014 and 2015. Preliminary data indicated the involvement of two distinct bacterial populations, one from San Diego and San Joaquin (SDi/SJo) and one from the San Francisco (SFr) Bay area. Whole-genome analysis and antibiotic susceptibility testing of 68 outbreak and archival isolates of *S. sonnei* were performed to investigate the microbiological factors related to these outbreaks. Both SDi/SJo and SFr populations, as well as almost all of the archival *S. sonnei* isolates belonged to sequence type 152 (ST152). Genome-wide single nucleotide polymorphism (SNP) analysis clustered the majority of California (CA) isolates to an earlier described lineage III. Isolates in the SDi/SJo population had a novel lambdoid bacteriophage carrying genes encoding Shiga toxin (STX) that were most closely related to that found in *Escherichia coli* O104:H4. However, the STX genes (*stx1A* and *stx1B*) from this novel phage had sequences most similar to the phages from *Shigella flexneri* and *S. dysenteriae*. The isolates in the SFr population were resistant to ciprofloxacin due to point mutations in *gyrA* and *parC* genes and were related to the fluoroquinolone-resistant *S. sonnei* clade within lineage III that originated in South Asia. The emergence of a highly virulent *S. sonnei* strain and introduction of a fluoroquinolone-resistant strain reflect the changing traits of this pathogen in California. An enhanced monitoring is advocated for early detection of future outbreaks caused by such strains.

**IMPORTANCE** Shigellosis is an acute diarrheal disease causing nearly half a million infections, 6,000 hospitalizations, and 70 deaths annually in the United States. *S. sonnei* caused two unusually large outbreaks in 2014 and 2015 in California. We used whole-genome sequencing to understand the pathogenic potential of bacteria involved in these outbreaks. Our results suggest the persistence of a local *S. sonnei* SDi/SJo clone in California since at least 2008. Recently, a derivative of the original clone acquired the ability to produce Shiga toxin (STX) via exchanges of bacteriophages with other bacteria. STX production is connected with more severe disease, including bloody diarrhea. A second population of *S. sonnei* that caused an outbreak in the San Francisco area was resistant to fluoroquinolones and showed evidence of connection to a fluoroquinolone-resistant lineage from South Asia. These emerging trends in *S. sonnei* populations in California must be monitored for future risks of the spread of increasingly virulent and resistant clones.

## Abstract (precis)

Shigellosis is an acute diarrheal disease causing nearly half a million infections, 6,000 hospitalizations, and 70 deaths annually in the United States. *S. sonnei* caused two unusually large outbreaks in 2014 and 2015 in California. We used whole-genome sequencing to understand the pathogenic potential of bacteria involved in these outbreaks. Our results suggest the persistence of a local *S. sonnei* SDi/SJo clone in California since at least 2008. Recently, a derivative of the original clone acquired the ability to produce Shiga toxin (STX) via exchanges of bacteriophages with other bacteria. STX production is connected with more severe disease, including bloody diarrhea. A second population of *S. sonnei* that caused an outbreak in the San Francisco area was resistant to fluoroquinolones and showed evidence of connection to a fluoroquinolone-resistant lineage from South Asia. These emerging trends in *S. sonnei* populations in California must be monitored for future risks of the spread of increasingly virulent and resistant clones.

## INTRODUCTION

Shigellosis is an acute gastrointestinal infection caused by bacteria belonging to the genus *Shigella*. Shigellosis is the third most common enteric bacterial infection in the United States with 500,000 infections, 6,000 hospitalizations, and 70 deaths each year (1). There are four *Shigella* species that cause shigellosis: *Shigella dysenteriae*, *S. flexneri*, *S. boydii*, and *S. sonnei* (2). *S. dysenteriae* is considered to be the most virulent species, particularly *S. dysenteriae* type 1 serotype due to its ability to produce a potent cytotoxin called Shiga toxin (STX). *S. flexneri*, *S. boydii*, and *S. sonnei* generally do not produce Shiga toxin and, therefore, cause mild forms of shigellosis (2, 3). Shiga toxins can also be produced by Shiga toxin-producing *Escherichia coli* (STEC). Two types of STX are known in STEC: STX 1 which differs by a single amino acid from STX in *S. dysenteriae* type 1, and STX 2 which shares only about 55% amino acid similarity with STX 1 (4). STX operons for both type 1 and 2 STX consist of the *stxA* and *stxB* subunit genes, which encode the AB_5_ holotoxin (5). Rarely, Shiga toxin *stx* genes can be transferred to non-STX-producing *S. flexneri* and *S. sonnei* by means of a lambdoid bacteriophage either from STEC or *S. dysenteriae* (3, 6, 7), providing those strains with the ability to cause more severe disease. Infections caused by bacterial species that produce STX often lead to hemorrhagic colitis and may cause serious complications, like hemolytic-uremic syndrome (HUS) (8). The epidemiology of different *Shigella* species also varies. *S. flexneri* is most common in developing countries (9), while *S. sonnei* is most common in developed countries (10, 11). *S. dysenteriae* is least common in the developed countries; however, it is often a cause of outbreaks in sub-Saharan Africa and South Asia (12). Infections caused by *S. boydii* are relatively uncommon worldwide (12). There is an emerging global trend of *S. sonnei* replacing *S. flexneri* in areas of developing countries undergoing economic growth and improvement in sanitation, such as Vietnam (13), and Bangladesh (14).

*S. sonnei* has caused two large outbreaks in California (CA) in 2014 and 2015. The first outbreak with distinct clusters in the San Diego and San Joaquin (SDi/SJo) areas was caused by STX-producing isolates (15). Another CA *S. sonnei* outbreak occurred within the same time frame as the two clusters described above, but it was caused by an STX-negative *S. sonnei* strain and was confined primarily to the San Francisco (SFr) Bay area (16). We performed whole-genome sequencing and antibiotic susceptibility testing of 68 outbreak and archival isolates to gain further insights into the microbiological factors associated with these shigellosis outbreaks. We examined the phylogeny of local *S. sonnei* isolates and also performed comparison of CA isolates with global *S. sonnei* clones. The results provided new insight into the likely origin of current CA *S. sonnei* isolates and evolutionary changes under way, as they acquire more virulence.

## RESULTS

### *In silico* MLST.

The sequences of seven housekeeping genes were extracted from genomic sequences from all isolates, and their allele numbers were identified using the Center for Genomic Epidemiology (CGE) multilocus sequence typing (MLST) database (17). The sequence type (ST) was assigned to each isolate based on the combination of identified alleles. All outbreak and archival California (CA) *S. sonnei* isolates had identical sequences for seven MLST loci and were assigned to the same sequence type, sequence type 152 (ST152), with the exception of one historical isolate C97 identified as ST1502. ST1502 differs from ST152 by a single allele. *S. sonnei* ST152 was previously found in Germany in 1997 and China in 2009 (18).

### High-quality SNP analysis.

**FIG 1:**
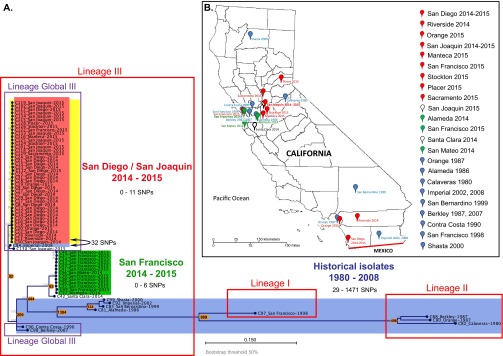
Local and global phylogeny of CA genome-wide hqSNP differences between STX1-producing *S. sonnei* isolates from the San Diego and San Joaquin outbreak, STX-negative *S. sonnei* isolates from the San Francisco outbreak, and historical isolates from California. Isolates from the San Diego/San Joaquin (SDi/SJo) outbreak (red), isolates from the San Francisco (SFr) outbreak (green), historical isolates (blue), and STX-positive isolates (yellow) are indicated by background colors. Major lineages I to III by Holt et al. (19) are outlined in red, and sublineage Global III by Holt et al. (19) is outlined in purple. Node labels are displayed as “Isolate ID_Geographic location in California–Date of isolation.” The numbers in orange circles or ovals correspond to the numbers of SNPs that are different between the closest isolates of the groups. (B) Geographical distribution of the CA *S. sonnei* isolates. The international boundary between the United States and Mexico is drawn in red. The colors of the pins correspond to the following groups of isolates: red, SDi/SJo population; green, SFr population; blue, historical isolates; white, modern sporadic isolates.

Based on genome-wide single nucleotide polymorphism (SNP) analysis, which is expected to provide better resolution of lineages than MLST, the current and archival isolates from California clustered into several clades (Fig. 1A). For genome-wide SNP analysis, high-quality SNPs (with coverage of ≥5×, minimum quality of >200, and minimum genotype quality of 10) were called against the reference in both coding and noncoding areas throughout the whole genome. All STX-positive isolates from two recent outbreak-related San Diego (SDi) and San Joaquin (SJo) epidemiological groups clustered together and differed from each other by 0 to 11 SNPs. The member of SDi epidemiological group C24 had 0 SNP differences with several SJo isolates (C101, C114 to C119, C122, C123, C125, C128, and C129), indicating direct relatedness of the isolates from the SDi and SJo epidemiological clusters. The only STX-negative isolate C130 from SJo had 48 SNP differences from the closest member of the STX-positive SDi/SJo group.

Isolates from the contemporary outbreak in San Francisco (SFr) formed a separate cluster (Fig. 1A) and had more SNP differences with the SDi/SJo cluster (251 SNPs) than with the historical isolate C96 from Contra Costa County, isolated in 1990 (219 SNPs). Isolate C42 from Santa Clara County (2014) was assigned to the SFr outbreak based on pulsed-field gel electrophoresis (PFGE) (see Data Set S1, sheet 1, in the supplemental material), but differed by 62 SNPs from the closest SFr outbreak member. Isolate C42 clustered closer to the SFr outbreak than to any other contemporary or historical isolate; therefore, it was considered to be a part of the SFr population.

10.1128/mSphere.00344-16.1 Data Set S1  (Sheet 1) List of California (CA) *S. sonnei* isolates. (Sheet 2) List of global *S. sonnei* strains used for phylogenetic comparison. (Sheet 3) Genes of representative SDi/SJo *S. sonnei* isolates with homologs in the closest *E. coli* strains, but not in other *S. sonnei* isolates. (Sheet 4) Antibiotic resistance genotypes and phenotypes of CA *S. sonnei* isolates. Download Data Set S1, XLSX file, 0.5 MB. Copyright © 2016 Kozyreva et al. 2016 Kozyreva et al. This content is distributed under the terms of the Creative Commons Attribution 4.0 International license.

The majority of historical isolates were genetically distinct from recent isolates except for isolate C84 from Imperial County (2008), which differed by 32 SNPs from the closest member of the SDi/SJo STX-positive cluster and by 28 SNPs from STX-negative SJo isolate C130. This is a surprisingly small number of SNPs, compared with the distance between the SDi/SJo cluster and other historical isolates (100 to 1,474 SNPs) or even to the contemporary SFr outbreak cluster (251 SNPs).

### Global phylogeny of CA *S. sonnei* isolates.

The genomes of CA *S. sonnei* isolates were compared with the genomes of global *S. sonnei* clones reported by previous studies (see Data Set S1, sheet 2, in the supplemental material) (19–21) using genome-wide SNP analysis. The isolates from the aforementioned studies (188 total) were selected based on following criteria: (i) to represent all lineages as per Holt et al. (19), (ii) different clades described by authors (Global III, Central Asia III), and (iii) various ciprofloxacin resistance mutations. The clustering of global strains in our analysis showed the same lineage patterns as in the original publications (Fig. S1). The current *S. sonnei* populations and the majority of the historical CA isolates (1986 to 2008) clustered with lineage III strains. All SDi/SJo population isolates as well as historical isolates C96, C98, and C84 (from 1990, 2007, and 2008, respectively) and recent STX-negative SDi isolate C130, clustered together with the Global III lineage. SDi/SJo clustered together with the historical isolate C84 from 2008 and the recent STX-negative C130 isolate, which were closely related and most likely represent a single introduction event dating back prior to 2008.

10.1128/mSphere.00344-16.2 Figure S1  Clustering of CA *S. sonnei* isolates with *S. sonnei* strains of global lineages based on a maximum likelihood phylogenetic tree built using genome-wide hqSNPs. Nodes are labeled with the name of country of isolation [isolates from this study are labeled USA (CA)]. The color of the node labels corresponds to a type of ciprofloxacin (CIP) resistance mutation. Branch and label colors correspond to the region of strain isolation. The lineages I to IV and Global III sublineage are outlined in gray based on lineage assignment by Holt et al. [19]). The sublineage outlined in yellow includes the South Asian clade, described previously (20), and the isolates that clustered with it. The tree is unrooted. The bootstrap display threshold is 75%. Download Figure S1, PDF file, 4 MB. Copyright © 2016 Kozyreva et al. 2016 Kozyreva et al. This content is distributed under the terms of the Creative Commons Attribution 4.0 International license.

Interestingly, historical isolate C98 from 2007 carrying fluoroquinolone (FQ) resistance mutation GyrA-83L clustered with a group of five genomes from Korea isolated between 1998 and 2003 carrying the same GyrA-83L mutation (19), which is not common within Global III lineage, suggesting a possible introduction route for the CA isolate from Korea in the past.

All ciprofloxacin-resistant *S. sonnei* isolates from the SFr outbreak with a triple mutation GyrA-83L GyrA-87G ParC-80I, clustered closely with the South Asia-Southeast Asia clade of isolates possessing the same type of FQ resistance mutation.

Another distinct clade within lineage III was formed by an isolate from Mexico (1998) and a group of historical CA *S. sonnei* isolates obtained between 1986 and 2002. Three CA isolates from 1980 to 1987 clustered together with lineage II. A single historical isolate C97, with a unique MLST pattern ST1502, was grouped with lineage I strains. No lineage IV isolates were found in California (Fig. 1A; see Fig. S1 in the supplemental material).

### COG and Pfam clustering of CA *S. sonnei* genomes.

Hierarchical clustering based on the COG (Clusters of Orthologous Groups of proteins) and Pfam (protein domain) profiles was used as an alternative method to examine relatedness of CA *S. sonnei* strains with other *S. sonnei* genomes in the Joint Genome Institute (JGI) Integrated Microbial Genomes (IMG) database and their phylogenetic position in relation to other *Shigella* species and *Escherichia coli* strains from the same database. This protein family profile clustering was carried out with representative genomes of CA *S. sonnei* isolates (isolates C7, 113, and C123 for the SDi/SJo population, isolate C39 for the SFr population, and isolates C84, C88, and C97 for historical isolates). We included all available *Shigella* genomes and all *E. coli* isolates that had a defined serovar in the JGI IMG database into the whole-genome comparison by Pfam and COG. Two different clustering methods were used: one based on the abundance and absence and the other based only on the presence/absence of both COG and Pfam groups.

The approach based on COG abundance showed all CA *S. sonnei* isolates clustering together with *E. coli* and separate from other *S. sonnei* isolates, with the exception of *S. sonnei* strain 1DT-1, which also clustered with *E. coli* (see Fig. S2A in the supplemental material). Similar results were seen in the hierarchical clustering based on Pfam abundance profiles (Fig. S2B). Except in the Pfam clustering, two CA *S. sonnei* isolates (one of the historical isolates and one San Francisco isolate) clustered with other *S. sonnei* isolates from the database, while all other CA isolates clustered with *E. coli*, as previously.

10.1128/mSphere.00344-16.3 Figure S2  Comparison of CA *S. sonnei* with other *Shigella* and *E. coli* species. (A) Hierarchical clustering of CA representative *S. sonnei* isolates with other *Shigella* and *E. coli* from JGI IMG database based on COG profiles (abundance). Representative *S. sonnei* isolates from California (red), other *S. sonnei* isolates from the JGI IMG database (green), and *E. coli* strains from the JGI IMG database (blue) are indicated. (B) Hierarchical clustering of CA representative *S. sonnei* isolates with other *Shigella* and *E. coli* isolates from the JGI IMG database based on Pfam profiles (abundance). Representative *S. sonnei* isolates from California (red), other *S. sonnei* isolates from the JGI IMG database (green), and *E. coli* strains from the JGI IMG database (blue) are indicated. (C) Comparison of CA *S. sonnei* with other *Shigella* and *E. coli* publicly available genomes based on nucleotide sequence. Maximum likelihood clustering from Phylosift nucleotide-based phylogeny was performed. *S. sonnei* isolates from California (red), other *S. sonnei* isolates from the JGI IMG and NCBI databases (green), and *E. coli* strains from JGI IMG database (blue) are indicated. The bootstrap threshold was 20%. (D) Comparison of CA *S. sonnei* with other *Shigella* and *E. coli* publicly available genomes based on nucleotide sequence. Maximum likelihood phylogeny of CA *S. sonnei*, *E. coli*, and publicly available *S. sonnei* genomes based on genome-wide hqSNPs was performed. *S. sonnei* isolates from California (red), other *S. sonnei* isolates from the JGI IMG and NCBI databases (green), and *E. coli* strains from the JGI IMG database (blue) are indicated. The bootstrap threshold was 50%. Download Figure S2, PDF file, 2.8 MB. Copyright © 2016 Kozyreva et al. 2016 Kozyreva et al. This content is distributed under the terms of the Creative Commons Attribution 4.0 International license.

To determine whether specific parts of genome were shared between CA *S. sonnei* and *E. coli* isolates and contributed to CA *S. sonnei* clustering with *E. coli*, we searched for the genes in the genome of CA *S. sonnei* that had homologs in either of two *E. coli* genomes which clustered closest to the CA *S. sonnei* on the COG tree (*E. coli* O104:H4 strain H112180541 and *E. coli* O139:H28 strain E24377A), but not in other *S. sonnei* isolates from the database (except for *S. sonnei* strain 1DT-1). The various genes (content and number) were found to be shared between either the *E. coli* strains and CA *S. sonnei* isolates, regardless of the presence of the STX bacteriophage in the latter case (see Data Set S1, sheet 3, in the supplemental material). When we searched for the genes that were common in all CA *S. sonnei* isolates and in one of the two closest *E. coli* strains, none were found. Thus, it was impossible to attribute the clustering of CA *S. sonnei* isolates with *E. coli* strains to the specific shared genes.

We also performed a recombination test on alignment of genome-wide high-quality SNPs (hqSNPs) using the Recombination Detection Program version 4 (RDP4) (22). None of the recombination detection algorithms included in the software package used detected recombination events between CA *S. sonnei* and *E. coli*.

To compare phylogenetic position of CA *S. sonnei* isolates in relation to other *Shigella* and *E. coli* species based on their nucleotide sequences, a phylogenetic tree of genomes was constructed from a concatenation of a set of 37 core phylogenetic marker genes using Phylosift (see Fig. S2C in the supplemental material) (23). We selected representative *E. coli* and *Shigella* species isolates from IMG JGI and NCBI databases sufficient to demonstrate clustering of CA isolates with one or another species and not attempting to show the phylogeny within each species. In this tree, all *S. sonnei* isolates, including CA *S. sonnei* isolates, grouped together, and separate from *E. coli* with one exception—*S. sonnei* strain 1DT-1 (which grouped in a clade with *E. coli*). Both nucleotide (Fig. S2C) and amino acid sequence (data not shown) trees generated with Phylosift showed the same result. Whole-genome clustering based on genome-wide SNPs in both coding and noncoding areas of the genome (Fig. S2D) supported the results of Phylosift: all *S. sonnei* isolates, including ones from California, clustered together, except for *S. sonnei* 1DT-1.

**FIG 2:**
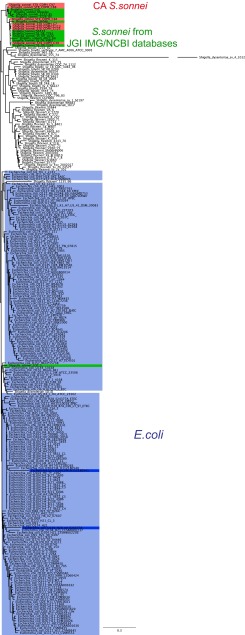
Comparison of CA *S. sonnei* isolates with other *Shigella* spp. and *E. coli* genomes**.** Revised maximum likelihood clustering of CA *S. sonnei* representative isolates with other *Shigella* species and *E. coli* isolates from the IMG JGI database based on COG profiles (presence/absence) is shown. Representative *S. sonnei* isolates from California (red), other *S. sonnei* isolates from the JGI IMG database (green), *E. coli* isolates from the JGI IMG database (blue), and two *E. coli* strains that previously clustered the closest to CA *S. sonnei* (dark blue) are indicated by background color.

The difference between protein family profile clustering and concatenated marker gene phylogeny is striking. In the protein family profile clustering, CA *S. sonnei* isolates grouped with *E. coli* rather than with other *S. sonnei*, but in the concatenated marker gene phylogeny, CA *S. sonnei* and *E. coli* did not group together. Closer examination of some of the results led us to consider the possibility that the observed contrast was an artifact of analysis. Upon further review, we found that CA isolates and *S. sonnei* strain 1DT-1 similar to *E. coli*, particularly serotype O104:H4, had low gene counts for several transposases (COG2963, COG2801, COG3547, COG3335, COG4584, and COG3385) and a DNA replication protein DnaC (COG1484), while other *Shigella* species isolates in the database had those genes in abundance (see Fig. S3 in the supplemental material). This indicated that a few transposable elements skewed the clustering of CA *S. sonnei* toward *E. coli*. When only the presence/absence of the COG and Pfam elements was taken into account, COG- and Pfam-based clustering appeared to be congruent with nucleotide phylogeny: CA *S. sonnei* isolates clustered with other *S. sonnei* strains in the database (Fig. 2; Fig. S4). Therefore, we would like to emphasize that genome clustering based on gene abundance is prone to the issues described above and should not be used to infer phylogenies.

10.1128/mSphere.00344-16.4 Figure S3  Comparison of COG abundance profiles of representative CA *S. sonnei* isolates with *E. coli* strains and other *Shigella* species from the JGI IMG database. The heat map shows the gene counts for different COGs. Download Figure S3, PDF file, 0.5 MB. Copyright © 2016 Kozyreva et al. 2016 Kozyreva et al. This content is distributed under the terms of the Creative Commons Attribution 4.0 International license.

10.1128/mSphere.00344-16.5 Figure S4  Revised Pfam phylogeny. Maximum likelihood clustering of CA representative *S. sonnei* isolates with *E. coli* and other *Shigella* species from the IMG JGI database based on Pfam profiles (presence/absence). Representative *S. sonnei* isolates from California (red), other *S. sonnei* isolates from the JGI IMG database (green), and *E. coli* strains from the JGI IMG database (blue) are indicated. Download Figure S4, PDF file, 1.2 MB. Copyright © 2016 Kozyreva et al. 2016 Kozyreva et al. This content is distributed under the terms of the Creative Commons Attribution 4.0 International license.

### STX-encoding bacteriophage from CA *S. sonnei* isolates*.*

**FIG 3:**
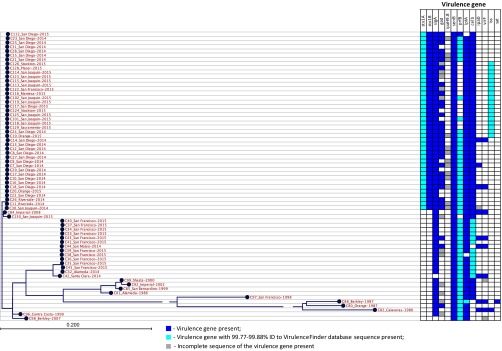
Virulence determinants in California *S. sonnei* isolates. Virulence determinants: *stx1A*, Shiga toxin 1, subunit A, variant a*; stx1B*, Shiga toxin 1, subunit B, variant a; *sigA*, *Shigella* IgA-like protease homolog*; gad*, glutamate decarboxylase*; ipaH9.8*, invasion plasmid antigen; *senB*, plasmid-encoded enterotoxin; *prfB*, P-related fimbriae regulatory gene; *lpfA*, long polar fimbriae; *celb*, endonuclease colicin E2; *ipaD*, invasion protein *S*. *flexneri*; *virF*, VirF transcriptional activator; *iss*, increased serum survival; *sat*, secreted autotransporter toxin.

All isolates from the SDi/SJo outbreak possessed STX-1 subunit A and B genes (*stx1A* and *stx1B*) (Fig. 3). Shiga toxin production by SDi/SJo isolates harboring *stx1* genes was confirmed in Vero cell neutralization assay. The data were presented in an earlier publication by Lamba et al. (15). None of the historical isolates or recent isolates related to the San Francisco outbreak had *stx* genes. The STX in the SDi/SJo population was encoded by a gene on a novel lambdoid bacteriophage. This 62.4-kb bacteriophage had identical sequence and was integrated into a chromosomal *wrbA* site in all SDi/SJo STX-positive isolates. The phage Ss-VASD isolated in California in 2016 from two *S. sonnei* isolates potentially related to the SDi/SJo outbreak cluster (24) was identical based on a BLASTn search to the bacteriophage characterized here. Due to unavailability of the isolates’ genome sequences, their relatedness to the outbreak cluster was impossible to confirm. A modern STX-negative *S. sonnei* isolate C130 from SJo had an intact *wrbA* site; therefore, we conclude that it represents a lineage sharing the STX-negative ancestor with the SDi/SJo population.

In order to better understand the origin of the novel STX phage in the SDi/SJo *S. sonnei* population, we carried out a series of comparative analyses. First, the Phage Search Tool (PHAST) revealed that the best match for the novel CA STX1 phage was the STX2-encoding P13374 phage found in STEC O104:H4, a strain that caused a large outbreak in Germany and other European countries in 2011 (25). The similarity of CA STX1 phage to STX phage from *E. coli* O104:H4 was supported by a BLASTn search, which revealed very high similarity with Stx2-encoding phages from *E. coli* O104:H4 strains 2009EL-2050 (CP003297.1) and 2009EL-2071 (CP003301.1) from Georgia in 2009 (25) with 98% shared identity at 92% query length. The bacteriophage from the CA isolates was also very similar to two phages found in strains from the European *E. coli* O104:H4 outbreak (P13374 phage [NC_018846]) with 97% identity for 88% of the query search coverage and the phage in *E. coli* strain 2011C-3493 (CP003289.1) with 97% identity for 93% of the query coverage. A recently characterized STX1-encoding *S. sonnei* phage 75/02_Stx from Hungary (KF766125.2) was also shown to share large colinear regions with STX2 prophages from *E. coli* O104:H4 and O157:H7 (5). However, the Hungarian phage only partially resembled CA STX1 phage with 99% of shared identity at 62% query coverage. Among other top BLASTn hits (≥97% identity [ID] and >90% query) were German *E. coli* HUSEC/EHEC (HUSEC stands for enterohemorrhagic *E*. *coli* [EHEC] associated with hemolytic-uremic syndrome [HUS]) epidemic isolate HUSEC2011 (HF572917.2) with 97% identity over 93% of the query, stx2-positive *E. coli* O168 strain 09-00049 from United States in 2009 (CP015228) with 97% ID/93% query, and another German outbreak *E. coli* O104:H4 strain C227-11 (CP011331.1) with 97% ID/92% query. The distance tree of the top BLASTn nucleotide sequences matches to the CA STX1 phage can be found in Fig. S5 in the supplemental material. The BLASTn distance tree was generated using all of the top hits regardless of species which had query cover of >70%, in addition to that, top hits among *Shigella* spp. with at least 30% query cover, and phages from *E. coli* O157:H7 strains Sakai and EDL933 were included in the comparison. This selection of phage sequences was further compared by several methods as described below.

10.1128/mSphere.00344-16.6 Figure S5  BLASTn distance tree of top matches to CA STX1 phage nucleotide sequence. The fast minimum evolution method of tree building was used. The tree is sorted by distance. The numbers over the branches designate branch length. The CA *S. sonnei* STX1 phage is indicated in yellow. Download Figure S5, PDF file, 0.8 MB. Copyright © 2016 Kozyreva et al. 2016 Kozyreva et al. This content is distributed under the terms of the Creative Commons Attribution 4.0 International license.

In order to better understand the phylogenetic relatedness of CA STX1 phage to other phages identified as top matches by BLASTn, we generated a phylogenetic tree of integrase genes. This revealed that the CA STX1 phage grouped evolutionarily with other STX-encoding *E. coli* phages, particularly with phages from non-O157 *E. coli*, but not with ones from *Shigella* species (see Fig. S6A in the supplemental material). Figure S7A shows the phylogeny based on integrase amino acid sequences; similar results were seen with nucleotide-based trees (not shown). This integrase phylogenetic analysis confirmed the relatedness of the CA STX1 phage to the phage found in *E. coli* O104:H4 from Georgia in 2009 and in pandemic *E. coli* O104:H4 from Germany in 2011, while it also showed similarity with two STX phages from *E. coli* O168:H8 isolated in the United States in 2009. It has been demonstrated previously that phage phylogeny should be inferred from a combination of protein repertoires and phage architecture rather than from a single gene (e.g., integrase) sequence (26, 27). The mosaic structure of the lambdoid phages poses a limitation for such single-locus phylogeny approach. For this reason, in addition to integrase phylogeny, we estimated full-phage-sequence phylogeny via multiple alignments of complete nucleotide sequences of the phages (Fig. S6B). The phylogeny based on complete phage sequences showed that phages from *E. coli* O104:H4 strains 2009EL-2050 and 2009EL-2071 from Georgia isolated in 2009 were the closest to the CA STX1 phage. The CA STX1 phage was also closely related to phages from other *E. coli* O104:H4 strains, including phage P13374 implicated in the German outbreak of 2011, and to the phage from STEC O2:H27 previously shown as highly similar to the German phage (25). To a lesser extent, the CA STX1 phage showed similarity to other phages from non-O157 strains (O168, O103, O111, and O26). STX phages from O157 strains of *E. coli* and other *Shigella* species were genetically distinct from the CA STX1 phage.

10.1128/mSphere.00344-16.7 Figure S6  Phylogeny of the CA STX1 phage. (A) Neighbor-joining phylogeny based on amino acid sequence of integrase protein. (B) Maximum likelihood phylogeny based on full nucleotide sequence of the phage. The CA STX1 phage is indicated in yellow. The numbers above the branches designate the bootstrap values. Isolates associated with European *E. coli* O104:H4 outbreak of 2011 are indicated in red. The color of the node corresponds to the type of Shiga toxin. Download Figure S6, PDF file, 0.7 MB. Copyright © 2016 Kozyreva et al. 2016 Kozyreva et al. This content is distributed under the terms of the Creative Commons Attribution 4.0 International license.

10.1128/mSphere.00344-16.8 Figure S7  Cryptic STX-converting prophages found in CA *S. sonnei* genomes. Download Figure S7, PDF file, 0.3 MB. Copyright © 2016 Kozyreva et al. 2016 Kozyreva et al. This content is distributed under the terms of the Creative Commons Attribution 4.0 International license.

**FIG 4:**
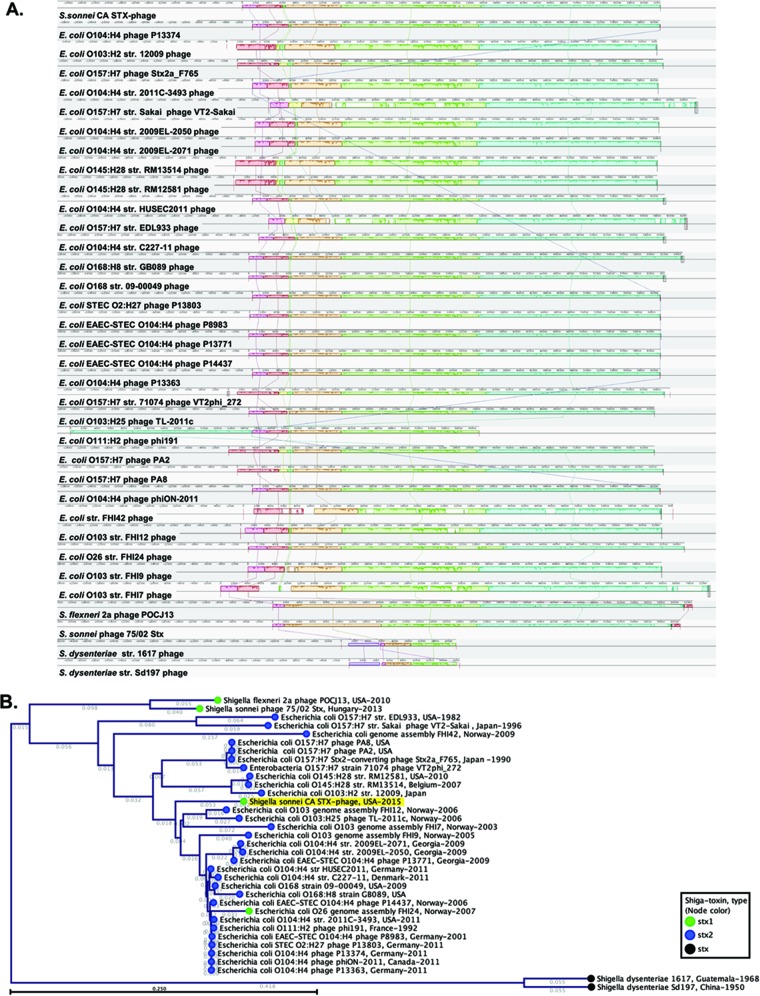
Phylogeny of Shiga toxin-encoding phage from SDi/SJo *S. sonnei* isolates. (A) progressiveMauve alignment of phage sequences. Sequences are centered by the *stxA* gene. str., strain. (B) A neighbor-joining tree based on an estimate of the shared gene content from the progressiveMauve alignment. The CA STX1 phage is indicated by yellow background color. The numbers over the branches designate branch length. The color of the node corresponds to the type of Shiga toxin.

We also used progressiveMauve to make whole-phage alignments. This revealed that the STX1 phage in CA isolates more closely resembled the STX2-coding phages in non-O157 *E. coli* than phages in O157 *E. coli* or *Shigella* species (Fig. 4A and B). The STX2-encoding phage P13374 from German outbreak *E. coli* O104:H4 strain, as well as related phages P13363 and phiON, were the closest to the CA STX1 phage according to the neighbor-joining tree built based on the estimate of the shared gene content from progressiveMauve alignment.

 We identified in several CA isolates the bacteriophages known to be associated with STX but that were missing actual *stx* genes. According to PHAST output, isolate C7 possessed an intact STX2-converting phage 1717 (NC_011357), isolate C113 had an incomplete STX2-converting phage 86 (NC_008464), and isolate C16 harbored a questionable STX2-converting phage I (NC_003525), while none of them contained the *stx* gene sequence (see Fig. S7 in the supplemental material). We propose gain and loss *stx* genes during the evolution of *S. sonnei* in California.

### STX holotoxin and other virulence determinants in CA *S. sonnei* isolates.

Even though the phage from *E. coli* O104:H4 STEC was identified as the most closely related to the CA STX1 phage, it carried the *stx2* gene, while the CA phage carried the gene encoding the STX1 toxin. The STX operon in CA isolates (holotoxin genes *stx1A* and *stx1B*) was identical to STX operons in *S. flexneri* phage POCJ13 (KJ603229.1) and several *S. dysenteriae* strains, including *S. dysenteriae* type 1 (M19437.1), but differed from STX operon in the majority of *E. coli* strains. The CA *S. sonnei* STX operon had 1 SNP difference with other STX1-encoding *S. sonnei* bacteriophages (accession nos. KF766125.2 and AJ132761.1). The similarity of different regions of the CA STX1 phage to various phages can be explained by the mosaic structure of STX lambdoid phages due to frequent recombinations and modular exchange with other lambdoid phages (28). Moreover, there is evidence that it could be quite common for lambdoid phages of *S. sonnei* to have *stx* genes from *S. dysenteriae* and the rest of the phage genes resembling the STEC or EHEC *E. coli* strains (3, 5).

Besides STX, the following virulence genes were detected in *S. sonnei* from California: *Shigella* IgA-like protease homolog (*sigA*), glutamate decarboxylase (*gad*), plasmid-encoded enterotoxin (*senB*), P-related fimbria regulatory gene (*prfB*), long polar fimbriae (*lpfA*), endonuclease colicin E2 (*celb*), invasion protein S. *flexneri* (*ipaD*), VirF transcriptional activator (*virF*), and increased serum survival gene (*iss*). The distribution of these virulence genes in different *S. sonnei* populations from California is presented in Fig. 3. The increased serum survival virulence gene *iss* was mainly found in the SDi/SJo population of isolates found more recently, and in a single historical isolate from 1980; however, the *iss* alleles in modern and historical isolates were different.

### Antimicrobial resistance of CA *S. sonnei* isolates.

**FIG 5:**
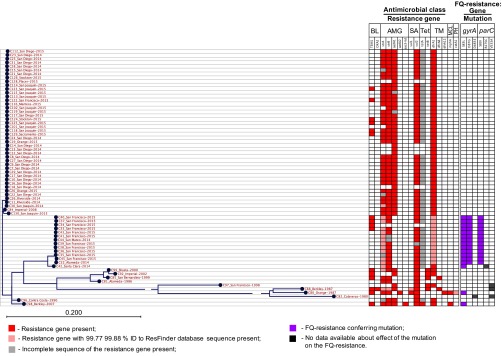
Antibiotic resistance determinants in CA *S. sonnei* isolates. Antimicrobial class abbreviations: BL, beta-lactams; AMG, aminoglycosides; SA, sulfonamides; Tet, tetracycline; TM, trimethoprim; MCL, macrolide; PH, phenicols; FQ, fluoroquinolones.

Acquired antibiotic resistance (ABR) markers to the following classes of antimicrobials were identified: beta-lactams (*bla*_TEM-1_ and *bla*_OXA-2_ genes), aminoglycosides [*aph*(*3*[dprime])*-Ib*, *aph*(*6*)*-Id*, *aac*(*3*)*-IId*, *aadA1*, and *aadA2*], macrolides (*mphA*), sulfonamides (*sul1* and *sul2*), phenicols (*catA1*), trimethoprim (*dfrA1*, *dfrA8*, and *dfrA12*), and tetracycline (*tetA and tetB*) (Fig. 5). The majority of the isolates were phenotypically resistant to aminoglycosides, sulfonamides, trimethoprim, and tetracycline. In addition, a phenotypic resistance to penicillins mediated by TEM-1 β-lactamase gene occurred in 16 isolates. The correlation between the genotype and susceptibility phenotype is presented in Data Set S1, sheet 4, in the supplemental material. In 100% of cases, the presence of antibiotic resistance determinants correlated with the expected nonsusceptible phenotype. In 7% of cases, the isolates without known antibiotic resistance genes appeared to be nonsusceptible (Data Set S1, sheet 4), suggesting the presence of resistance mechanisms, like the loss of porins and increased efflux (29), which are not included in the ResFinder database. For example, two isolates showed intermediate susceptibility to streptomycin, and 15 isolates were nonsusceptible to ampicillin and sulbactam in the absence of the genes which would explain the corresponding resistance phenotypes in those isolates. Among the tetracycline-resistant isolates, 3.3% (*n* = 2) of isolates did not possess any known tetracycline resistance genes, while 86.9% (*n* = 53) had a truncated version of the *tet*(A) gene (1,172 bp versus 1,200 bp for the full-length reference sequence from the ResFinder database). Nonetheless, the isolates without tetracyclin resistance genes or with incomplete *tet*(A) gene demonstrated a high level of resistance to tetracycline (MIC > 8 μg/ml). This suggests the presence of additional mechanisms of resistance that were not detected in the ResFinder database.

Acquired ABR genes were frequently associated with various mobile elements. Penicillin resistance encoded on the *bla*_TEM-1_ gene was associated with Tn*3* transposon located on conjugative plasmids of the IncB/O/K/Z incompatibility group found in both recent SFr and historical isolates (see Fig. S8A in the supplemental material) or on the putative IncI1 plasmid in several modern SJo isolates (Fig. S8B). Partial plasmid sequences were identified in historical isolates as IncB/O/K/Z and contained plasmid backbone genes encoding the following products: transcriptional activator RfaH, plasmid stabilization system protein, ribbon-helix-helix protein CopG, replication initiation protein RepA, and plasmid conjugative transfer protein. These partial IncB/O/K/Z plasmid sequences in historical isolates matched the analogous area in IncB/O/K/Z plasmids of recent isolates, suggesting their relatedness to each other. An identical genetic element Tn*3*::*bla*_TEM-1_ was integrated into different loci of the IncB/O/K/Z plasmids in historical isolates in comparison with integration locus in IncB/O/K/Z plasmids of modern San Francisco cluster isolates, which suggests that dissemination of the *bla*_TEM-1_ gene is associated with the transfer via Tn*3* (Fig. S8A).

10.1128/mSphere.00344-16.9 Figure S8  Plasmids and other mobile elements encoding antibiotic resistance in CA *S*. *sonnei*. (A) Organization of *bla*_TEM-1_-carrying IncB/O/K/Z conjugative plasmid from modern SFr population isolates. Different integration sites of Tn*3*::*bla*_TEM-1_ on IncB/O/K/Z plasmids found in recent SFr isolates and in historical *S. sonnei* isolates. (B) A representative genetic region surrounding the *bla*_TEM-1_ gene on a putative IncI1 conjugative plasmid from a modern SJo *S. sonnei* isolate. (C) Representative organization of the *sul2-strA-strB-tetA* resistance gene cluster in SDi/SJo and historical CA *S. sonnei* isolates. (D) Genetic region surrounding the macrolide resistance gene in historical *S. sonnei* isolate C80 (Orange County, 1987). Abbreviation: hp, hypothetical protein. Download Figure S8, PDF file, 1.6 MB. Copyright © 2016 Kozyreva et al. 2016 Kozyreva et al. This content is distributed under the terms of the Creative Commons Attribution 4.0 International license.

Aminoglycoside and trimethoprim resistance genes were often linked with Tn*7* transposon (see Fig. S8C in the supplemental material). A genetic region, containing an association of Tn*7* with *aadA1* and *dfrA1* ABR genes, as well as TDP-fucosamine acetyltransferase, and a partial sequence of tyrosine recombinase XerC/D were identical in recent SJo isolates and two historical samples C99 and C92, isolated in Shasta County in 2000 and Imperial County in 2002, respectively. However, the area upstream of Tn*7* in novel isolates showed evidence of later integration of an additional Tn*7* element, leading to the loss or modification of the area containing tetracycline resistance genes found in historical isolates.

One of the historical isolates C80 (Orange County, 1987) was resistant to azithromycin and possessed macrolide resistance gene *mphA*, found in association with Tn*7* transposon on a contig which also encoded trimethoprim, sulfamethoxazole, and aminoglycoside resistance, multidrug transporter EmrE, and a mercuric resistance operon (see Fig. S8D in the supplemental material). Resistance to azithromycin is emerging in the United States (30), and the presence of an azithromycin resistance gene in one of the isolates dating back to 1987 is remarkable.

### Fluoroquinolone resistance.

All modern isolates from the SDi/SJo population and historical *S. sonnei* isolates were susceptible to fluoroquinolones (FQ); however, few historical isolates possessed several types of point mutations leading to amino acid substitutions in the genes encoding FQ targets, such as the A subunit of DNA gyrase (GyrA) and the C subunit of topoisomerase IV (ParC) (Fig. 5; see Data Set S1, sheet 4, in the supplemental material). The quinolone resistance-determining region (QRDR) (31) of the GyrA in two CA historical isolates had a single amino acid substitution S83L. *S. sonnei* isolates with single mutation GyrA-S83L have been previously reported to exhibit high MICs for quinolones (nalidixic acid) but low MICs for fluoroquinolones (MIC range of ciprofloxacin, 0.125 to 0.25 μg/ml) (32). In agreement with previous data, historical CA *S. sonnei* isolates with single mutation GyrA-S83L remained phenotypically susceptible to ciprofloxacin with wild-type MIC values of ≤0.5 μg/ml. Two FQ-susceptible historical isolates had a single point mutation V533A in the ParC, and one FQ-susceptible isolate had a combination of ParC-V533A and GyrA-D483E mutations; none of the mutations were located in the QRDRs of corresponding proteins and were not associated with a resistant phenotype.

In contrast, all SFr population isolates implicated in the outbreak expressed high-level FQ resistance. All isolates from the contemporary San Francisco outbreak had a combination of point mutations S83L and D87G in QRDR GyrA and substitution S80I in QRDR ParC, which caused a significant increase in ciprofloxacin MIC to ≥4 μg/ml (see Data Set S1, sheet 4, in the supplemental material). The modern nonoutbreak isolate C42 was phenotypically susceptible to FQ and possessed a single QRDR GyrA mutation S83L (Fig. 5), which represents a prerequisite mutation allowing for one-step development of high-level FQ resistance if combined with another QRDR mutation (33).

## DISCUSSION

The key results of our study were as follows: (i) the first large-scale whole-genome analysis of *S. sonnei* strains from North America; (ii) the delineation of two distinct populations of *S. sonnei* that caused large outbreaks of shigellosis in California (SDi/SJo population and SFr population); (iii) linkage of SDi/SJo population to an older lineage of *S. sonnei* documented in California as early as 2008; (iv) evidence of STX1-encoding bacteriophage in the SDi/SJo population related to the phages from *E. coli* O104:H4, including a pandemic strain from Germany with the STX operon identical to those in *S. flexneri* and *S. dysenteriae*; (v) connection of the SFr population to an FQ-resistant clade that originated in South Asia; (vi) evidence of fluoroquinolone resistance in the SFr population mediated by the chromosomal mutations in the genes of antibiotic targets GyrA and ParC.

According to the whole-genome hqSNP phylogeny, the isolates from outbreaks in 2014 to 2015 were divided into two distinct populations—SDi/SJo and SFr. SDi and SJo isolates constituted the same outbreak population, which was incongruent with two separate clusters identified by PFGE (15). Many recent publications have highlighted superior discriminatory power and predictive value of whole-genome sequencing (WGS) over PFGE for genotyping of bacterial strains (34).

Comparison to global *S. sonnei* clones placed both modern SDi/SJo and SFr populations as well as the majority of historical CA *S. sonnei* isolates dating back as far as 1986 into the lineage III defined by Holt et al. (19) (Fig. 1A; see Fig. S1 in the supplemental material). All STX-positive SDi/SJo isolates clustered with Global III clade defined by Holt et al. within lineage III, which was shown to be particularly successful at global expansion (19). Remarkably, the isolates from the SDi/SJo population did not cluster with geographically proximate isolates from Mexico, which was related to only a few historical CA isolates, suggesting that the strain exchange with Mexico had happened in the past, but is unlikely to be a source of recent introduction of STX-producing *S. sonnei* into California. According to the Holt et al. hypothesis, all original *S. sonnei* lineages diversified from Europe and then were introduced to other countries where they underwent localized clonal expansions (19). Our findings suggest that the lineage ancestral to the current SDi/SJo *S. sonnei* population was introduced as early as 2008 and diversified within California. The data suggest that the acquisition of the *stx1* gene happened after the introduced Global III lineage *S. sonnei* clone had already spread in California. The contemporary C130 and historical C84 STX-negative *S. sonnei* isolates, which both clustered with STX-positive SDi/SJo outbreak isolates, most likely represent the closest lineage to an ancestral STX-negative strain from which the SDi/SJo population evolved by acquisition of the STX bacteriophage.

All ciprofloxacin-resistant *S. sonnei* isolates from the SFr population showed close clustering with the South Asia-Southeast Asia clade of FQ-resistant isolates. South Asia has been shown to serve as a hub for dissemination of FQ-resistant *S. sonnei* clones around the globe (20), and our data suggest that the CA FQ-resistant SFr population also originated from the South Asian clade. Considering that no patients from the SFr outbreak cluster reported international travel (16), the introduced resistant clone could have been circulating locally prior to involvement in the outbreak. Unlike some of the other U.S. isolates deposited as a part of GenomeTrakr project, CA isolates did not cluster with European FQ-resistant isolates and likely represent a direct introduction event. As *S. sonnei* is becoming more common in developing countries (13, 14), as in many of the countries in South and Southeast Asia, it could further increase the risk of clonal expansion and global dissemination of FQ-resistant strains of *S. sonnei*.

It also appears that global *S. sonnei* clones belonging to lineages I, II, and III were introduced into California independently during the last 3 decades.

All recent *S. sonnei* outbreak isolates as well as all but one historical isolate belonged to ST152. This long-term persistence of a single clone in California over 3 decades is remarkable, and it could indicate that ST152 is a very successful *S. sonnei* clone. There were previous examples when the same sequence types persisted in the same geographical area for a long time, e.g., ST245 in China was found in 1983 as well as 26 years later in 2009 (18). This phenomenon, however, is most likely explained by a low resolution of MLST for subtyping of clonal *S. sonnei* population, which has been noted before (35).

Isolates from the SDi/SJo *S. sonnei* population, which caused outbreak of severe diarrheal disease (15), carried *stx1* genes carried on a novel lambdoid bacteriophage in all of the isolates. The strains of *E. coli* O104:H4 serotype appeared to be the most likely source of the STX bacteriophage in SDi/SJo *S. sonnei* isolates based on overall similarity of their whole-phage sequences. Specifically, the STX2 phage from *E. coli* O104:H4 strain isolated in Georgia in 2009 was found to be the closest known relative to CA STX1 phage. This Georgian strain was previously shown to share a common ancestry with *E. coli* O104:H4 strain implicated in an outbreak in Germany and other European countries in 2011 (36), one of the world’s largest outbreaks of food-borne disease in humans with 855 cases of hemolytic-uremic syndrome (HUS) and 53 fatalities (37, 38). It has been also suggested that the STX bacteriophage found in Georgian isolates resided in the common ancestor of Georgian and German outbreak strains (36). It is possible that CA *S. sonnei* received the STX bacteriophage from *E. coli* O104:H4 strain related to Georgian isolates or from the strain that served as a parent to the Georgian and German outbreak strains. The STX phage from *E. coli* O104:H4 has overcome recombination of the STX2 operon to yield the final phage with general architecture of *E. coli* phage and STX1 operon from *S. flexneri* or *S. dysenteriae*.

STX1-encoding bacteriophage was not found in the contemporary isolate C130 even though it was closely related to SDi/SJo *S. sonnei* population. It is possible that the bacterial population as a whole acquired the STX1-encoding bacteriophage more recently and concurrently. Such a scenario would also suggest that the increase in virulence can occur either by the spread of STX-positive clones or by the horizontal transfer of *stx1* genes by bacteriophages. The potential of bacteriophages to transfer STX genes from one *S. sonnei* strain to another has been described previously (3). Not all the *S. sonnei* strains have a suitable genetic background to be able to express STX efficiently (39). The modern STX-positive SDi/SJo population of *S. sonnei* was shown to be able to express STX (15) and thus has been proven to possess the genetic background that is adapted for increased virulence. The adaptation of SDi/SJo *S. sonnei* genomic background to retain and express STX stably by itself is a prerequisite for future emergence of more virulent strains in California. Another example of SDi/SJo population increasing its virulence is the acquisition of the increased serum survival virulence gene *iss*, which occurred seemingly recently in the evolution of the SDi/SJo *S. sonnei* clone. To our knowledge, this is the first report of the increased serum survival determinant (*iss*) being found in *Shigella* species.

Even though the treatment of infection caused by *S. sonnei* with antibiotics is not a standard procedure, it is indicated for the treatment of severe cases in order to reduce duration of symptoms (40). Antibiotic resistance (AR) of *S. sonnei* is, however, on the rise (16, 41, 42). This highlights the importance of ABR monitoring in *S. sonnei* populations. We detected resistance markers to multiple classes of antimicrobials in CA *S. sonnei*. For example, 91.2% of modern isolates and 81.8% of historical isolates were multidrug resistant (MDR) according to the definition of MDR microorganisms as demonstrating “non-susceptibility to at least one agent in three or more antimicrobial categories” (43). Acquired ABR genes in CA *S. sonnei* were frequently associated with various mobile elements and conjugative plasmids, likely contributing to their dissemination. For example, MDR transposon Tn7 has been shown to be a crucial part of ABR evolution in the local populations of *S. sonnei* lineage III (19). Once acquired, ABR genes tend to stay in the bacterial population, e.g., traditional antibiotics like co-trimoxazole and tetracycline are no longer in use for patient treatment (18), but the ABR genes persist in the modern SDi/SJo and SFr populations of the CA *S. sonnei*. Due to excessive veterinary use of antibiotics, farm animals were known to be a significant contributor of antibiotic resistance in enteric pathogens (44), like *S. sonnei*. Therefore, the development and persistence of multidrug resistance in CA *S. sonnei* could be attributed to the possible circulation of the pathogen in an animal host. However, since no point source or common exposures were identified for either SFr (16) or SDi/SJo (15) outbreaks, it is difficult to determine the conditions leading to the evolution of the strains. The multiple secondary cases of SDi/SJo outbreak caused by person-to-person transmission (15) and extended duration of the outbreak raised the possibility of lengthy circulation of CA *S. sonnei* in the human population.

Particularly worrisome was the detection of FQ resistance in all of the isolates belonging to a modern SFr *S. sonnei* population implicated in the outbreak. FQ resistance in all SFr outbreak isolates was mediated by a combination of double mutations in the QRDR GyrA of DNA gyrase and one mutation in QRDR ParC of topoisomerase IV. All historical isolates and all other modern isolates were susceptible to FQ; however, few of those phenotypically susceptible CA *S. sonnei* isolates possessed a prerequisite single amino acid substitution in QRDR of the GyrA, including two isolates belonging to CA lineage III: one historical isolate from 2007 and one modern nonoutbreak isolate belonging to the SFr population. Single mutations in QRDR GyrA were shown previously to confer a low level of FQ resistance and to serve a prerequisite for further resistance escalation via stepwise mutation acquisition (33). Therefore, emergence and dissemination of such low-FQ-resistance strains should be a concern for public health authorities.

We acknowledge certain limitations of this study. The temporal and spatial representation was sporadic in *S. sonnei* strains in our culture collection. We also recognize that the short-read sequencing by synthesis used to generate data might limit complete assembly of *S. sonnei* genomes and enumeration of genome-wide SNPs. No plasmid transformation followed by resequencing could be performed to confirm postulated genetic exchanges among *S. sonnei* populations.

Although the direct experimental evidence is lacking, Shiga toxin production and other virulence elements discovered in the SDi/SJo population appeared to be among the contributors that lead to serious manifestations of gastrointestinal disease in the CA shigellosis outbreak, including bloody diarrhea in 71% of patients (15). Fortunately, there were no fatalities and none of the affected patients developed the more-serious HUS. One potential clue to the absence of severe manifestations could be that Shiga toxin-positive *S. sonnei* strains contained STX1. The toxins encoded by STX1 and STX2 are known to elicit variable pathology among affected individuals. A number of investigators have demonstrated that *E. coli* O157 Shiga toxin-producing strains carrying STX2 caused more-severe disease, including HUS, than STX1-positive strains (45–47).

### Conclusions. 

Two distinct populations of *S. sonnei* (SDi/SJo and SFr) were delineated in the recent shigellosis outbreaks in California. The SDi/SJo population evolved from a lineage of *S. sonnei* likely present in California as early as 2008. The suggested evolutionary pathway of the SDi/SJo population was enhanced virulence via acquisition of a phage from the *E. coli* O104:H4 strain and STX operon from *S. flexneri* or *S. dysenteriae*. Close relatedness of the fluoroquinolone-resistant SFr population to the *S. sonnei* clade from South and Southeast Asia harboring mutations in the *gyrA* and *parC* genes suggested the possibility of foreign introduction of this clone into California. Thus, the recent outbreak *S. sonnei* strains in California are characterized by (i) acquisition of increased virulence by a local clone and (ii) introduction of a fluoroquinolone-resistant clone from abroad. Both trends are of a concern to public health authorities and call for enhanced surveillance and early detection of future shigellosis outbreaks.

## MATERIALS AND METHODS

### Isolates.

Sixty-eight *Shigella sonnei* human isolates from California (CA) (57 outbreak-related isolates from 2014 to 2015 and 11 archival isolates from 1980 to 2008), were identified and serotyped by standard methods (48) (see Data Set S1, sheet 1, in the supplemental material). PCR detection of the *stx*_1_ and *stx*_2_ genes and Vero cell neutralization assay for confirmation of STX production were performed as previously described (49). A map with geographical distribution of the locations of origin of the isolates is shown in Fig. 1B.

### Whole-genome sequencing (WGS) and data analysis.

DNA was extracted with a Wizard Genomic DNA kit (Promega, Madison, WI). Sequencing libraries were constructed using the Nextera XT (Illumina Inc., San Diego, CA) library preparation kit. Sequencing was performed using 2 × 300-bp sequencing chemistry on an Illumina MiSeq sequencer per the manufacturer’s instructions. Adapter trimming and demultiplexing were performed using MiSeq Reporter software. Prior to data analysis, all reads were quality trimmed to remove all ambiguous nucleotides and sequences with a Phred score of <30, and to discard all reads of <40 bp using CLCbio Genomic Workbench 8.0.2 (Qiagen, Aarhus, Denmark).

High-quality single nucleotide polymorphism (hqSNP)-based phylogeny was used to determine genetic relationships between the local California (CA) *S. sonnei* populations and their connection to global *S. sonnei* strains. See the lists of local isolates and global strains used in the analysis in sheets 1 and 2, respectively, in Data Set S1 in the supplemental material. The details of hqSNP analysis can be found in File S1. In general, paired-end reads were mapped to the reference genome of *S. sonnei* Ss046 (NC_007384.1) using CLCbio Genomic Workbench 8.0.2 (Qiagen, Aarhus, Denmark). SNPs were called in coding and noncoding genome areas using SAMtools mpileup (v.1.2 [50]), BCFtools (v 0.1.19; http://samtools.github.io/bcftools/), VCFtools (v.0.1.12b [51]). Only high-quality single nucleotide polymorphisms were included using the following criteria: coverage of ≥5×, minimum quality of >200, minimum genotype quality (GQ) of 10 (–minDP 5; –minQ 200; –minGQ 10; –remove-indels), without indels and the heterozygote calls. A phylogenetic tree was generated using CLCbio Genomic Workbench 8.0.2 (Qiagen, Aarhus, Denmark) with maximum likelihood phylogeny (under the Jukes-Cantor or general time reversible nucleotide substitution models, as specified; with bootstrapping) based on hqSNPs.

10.1128/mSphere.00344-16.10 Text S1  Detailed description of high-quality SNP (hqSNP)-based phylogenetic analysis. Download Text S1, PDF file, 0.1 MB. Copyright © 2016 Kozyreva et al. 2016 Kozyreva et al. This content is distributed under the terms of the Creative Commons Attribution 4.0 International license.

Clustering of genomes based on COG and Pfam profiles and using Phylosift (23) was performed to compare CA *S. sonnei* strains with other *S. sonnei* genomes found in JGI IMG database and show phylogenetic position of CA isolates and other *S. sonnei* strains in relation to other *Shigella* species and *Escherichia coli* strains in the same database.

COG- and Pfam-based identification and clustering were performed using the Department of Energy (DOE) Joint Genome Institute (JGI) Integrated Microbial Genomes (IMG) system (https://img.jgi.doe.gov/). The presence or absence matrices were fed to RAxML 7.3.0 (52) to produce the best tree from 50 bootstraps using the gamma model rate of heterogeneity for binary input. COG abundance profile and gene homolog search were performed using JGI IMG tools.

Phylosift (23) was used to (i) identify 37 “universal” genes in a set of genomes (including those generated here) and (ii) generate alignments for each gene family and then concatenate the alignments. A phylogenetic tree was inferred from the concatenated alignments using RAxML 7.2.6. Bipartition trees were generated using 1,000 bootstraps.

*De novo* assembly for each genome was done on CLCbio GW8.0.2. The assembled genomes of *S. sonnei* isolates had 30× to 159× sequencing coverage. Genomes were annotated with prokka v1.1 (53), the JGI IMG database, the Center for Genomic Epidemiology (CGE) (ResFinder, VirulenceFinder, PlasmidFinder) (54), and the Phage Search Tool (PHAST) (55) online resources. *In silico* multilocus sequence typing (MLST) was performed using the CGE online tool (17) against the MLST scheme “Escherichia coli #1” (https://cge.cbs.dtu.dk/services/MLST/) (56).

The primary search for the bacteriophage sequences similar to the CA STX1 phage was performed using BLASTn and Phage Search Tool (PHAST). To estimate similarity between the bacteriophages, sequence alignment and a neighbor-joining tree (based on the estimate of the shared gene content) were generated using the progressiveMauve program (57), and in addition, the full phage sequences were aligned using CLCbio GW8.0.2. general aligner, and a maximum likelihood tree was generated under the Jukes-Cantor nucleotide substitution model with bootstrapping. Sequences of integrases (both the DNA for the genes and the encoded amino acids for the proteins), derived from the sequences of STX phages were aligned using CLCbio GW8.0.2 general aligner, and a neighbor-joining tree was generated as described above.

Recombination test was performed on genome-wide hqSNP alignment using Recombination Detection Program version 4.67 (RDP4) (22). The following algorithms included in the RDP4 package were applied to search for the recombination events: RDP, BOOTSCAN, GENECONV, MAXCHI, CHIMAERA, SISCAN, 3SEQ, PHYLPRO, and VisRD. A window size of 100 and a step size of 30 were used.

### Antimicrobial susceptibility and resistance testing.

Antimicrobial susceptibility testing of *S. sonnei* isolates was performed using Microscan Dried Gram-negative panels Neg MIC 38 (Beckman Coulter, Inc., Brea, CA, USA); the MIC results were read and interpreted according to the manufacturer’s instructions. Streptomycin (10 μg) and azithromycin (15 μg) BBL Sensi-Discs (Becton, Dickinson, Franklin Lakes, NJ, USA) were used to determine susceptibility to the corresponding antimicrobials. Standard quality control strains were tested in parallel as required in respective product inserts.

### Accession number(s).

This whole-genome shotgun project has been deposited at GenBank under accession numbers LXUL00000000 to LXVV00000000, LYDX00000000 to LYEW00000000, and LRRZ00000000 to LRSD00000000. The version described in this paper is version LXUL01000000 to LXVV01000000, LYDX01000000 to LYEW01000000, and LRRZ01000000 to LRSD01000000. The raw reads for samples sequenced here were submitted to NCBI SRA archive under GenBank accession number SRP073631.
