# Supplementary material for: Recent Outbreaks of Shigellosis in California Caused by Two Distinct Populations of Shigella sonnei with either Increased Virulence or Fluoroquinolone Resistance
Source: mSphere. 2016 Dec 21;1(6):e00344-16. doi: 10.1128/mSphere.00344-16 (PMC5177732; doi:10.1128/mSphere.00344-16)

Figure S1. Clustering of CA *S. sonnei* isolates with *S. sonnei* strains of global lineages based on a Maximum Likelihood phylogenetic tree built using genome-wide hqSNPs.
Nodes are lebeled with name of country of isolation (isolates from this study are labeled as USA (CA)). Color of node labels corresponds to a type of CIP-resistance mutation. Branch and label colors correspond to the region of strain isolation. The lineages I-IV and Global III sublineage are framed with grey line (based on lineage assignment by Holt et al.). The sublineage highlighted with yellow frame includes South Asian clade, described previously, and isolates which clustered with it. Tree is unrooted. Bootstrap display threshold is 75%.

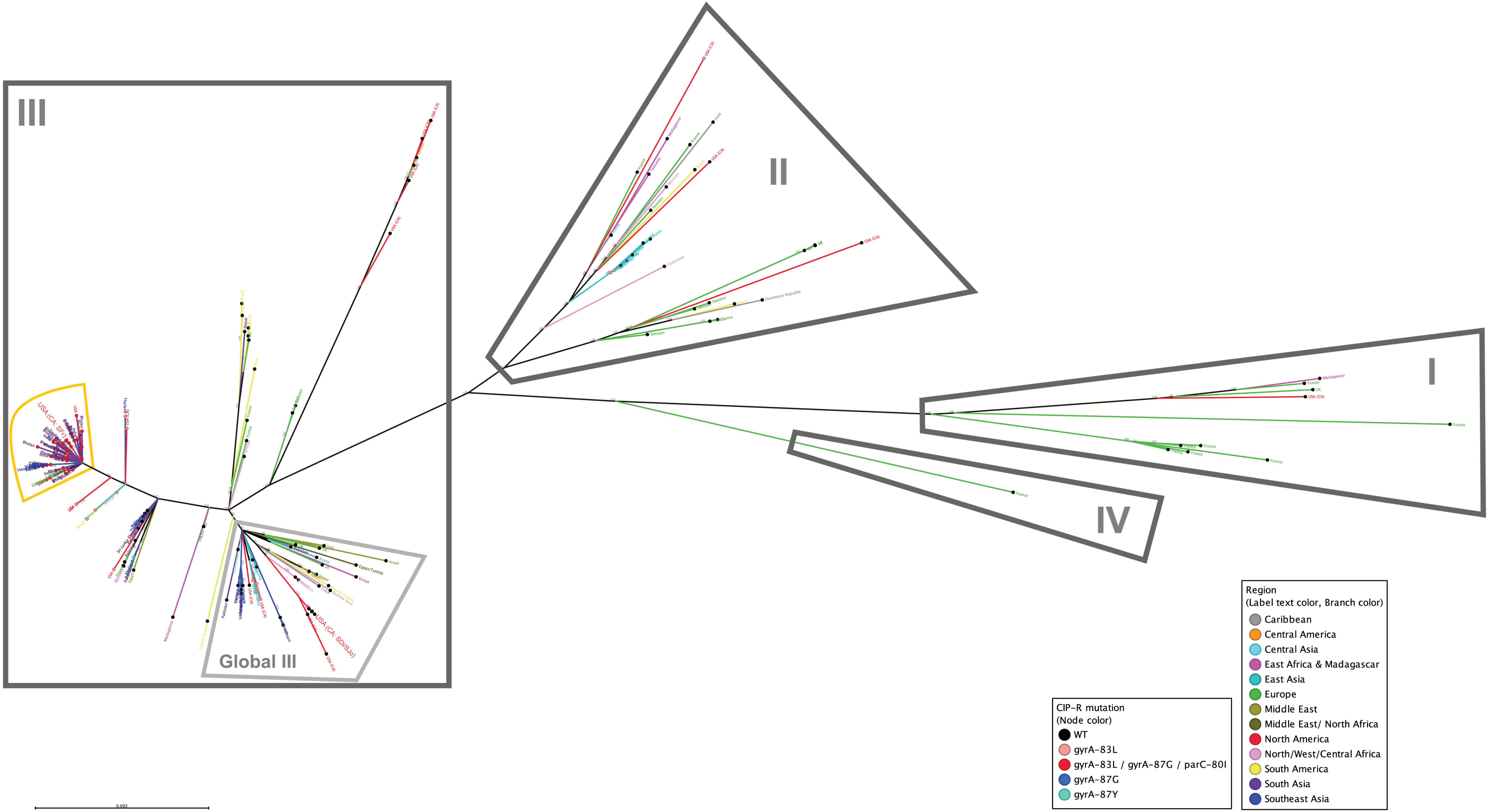

Supplement: Figure S1 [file sph006162211sf2.pdf]
