# Supplementary material for: Recent Outbreaks of Shigellosis in California Caused by Two Distinct Populations of Shigella sonnei with either Increased Virulence or Fluoroquinolone Resistance
Source: mSphere. 2016 Dec 21;1(6):e00344-16. doi: 10.1128/mSphere.00344-16 (PMC5177732; doi:10.1128/mSphere.00344-16)

Figure S2. Comparison of CA *S. sonnei* with other *Shigella* and *E. coli* species

A. Hierarchical Clustering of CA representative *S. sonnei* isolates with other *Shigella* and *E. coli* from JGI IMG database based on COG profiles (abundance). Color of the brackets: Red- representative *S. sonnei* from California; Green- other *S. sonnei* from JGI IMG database; Blue- *E. coli* strains from JGI IMG database.

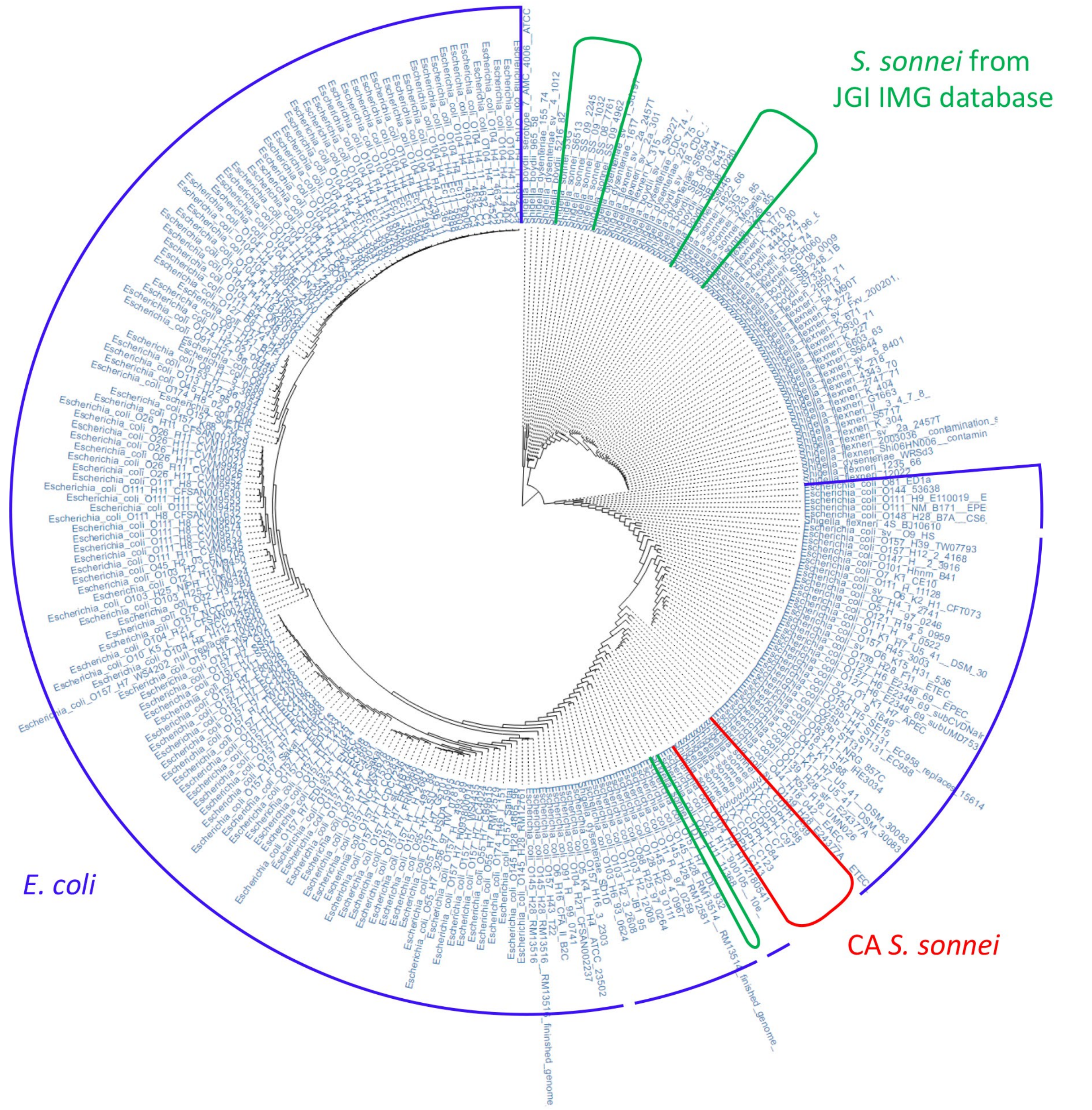

B. Hierarchical Clustering of CA representative *S. sonnei* isolates with other *Shigella* and *E. coli* from JGI IMG database based on pfam profiles (abundance). Color of the brackets: Red- representative *S. sonnei* from California; Green- other *S. sonnei* from JGI IMG database; Blue- *E. coli* strains from JGI IMG database.

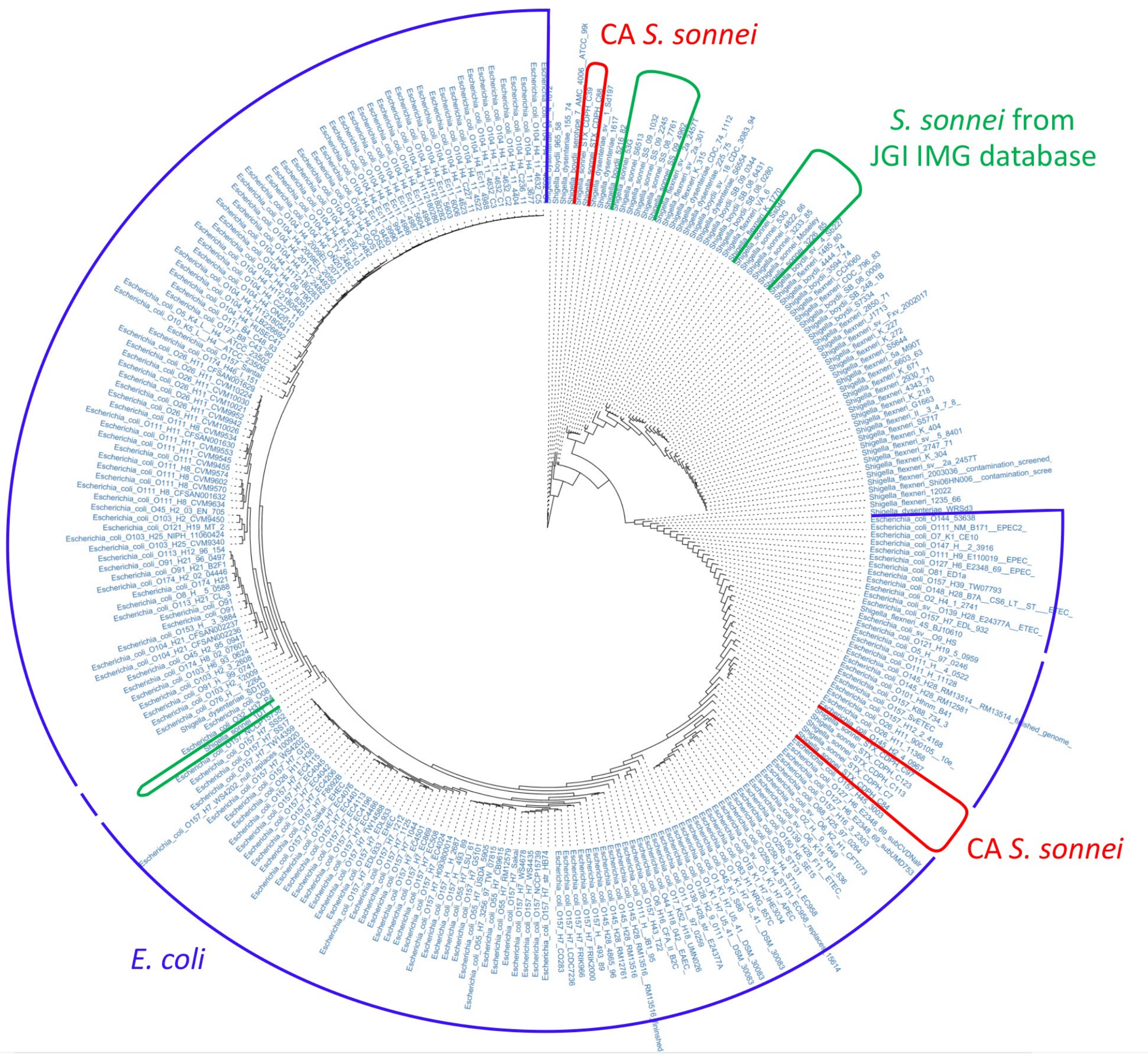

C. Comparison of CA *S. sonnei* with other *Shigella* and *E. coli* publicly-available genomes based on nucleotide sequence. Maximum likelihood clustering from PhyloSift nucleotide-based phylogeny. Background color: Red- *S.sonnei* from California; Green- other *S.sonnei* from JGI IMG and NCBI databases; Blue- *E.coli* strains from JGI IMG database. Bootstrap threshold 20%.

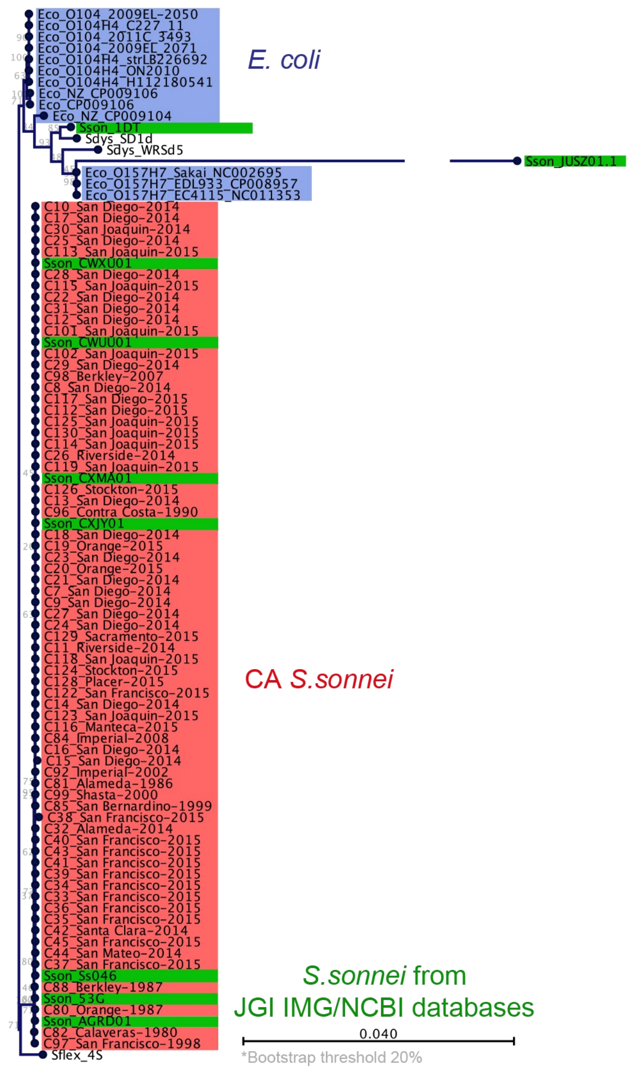

D. Comparison of CA *S. sonnei* with other *Shigella* and *E. coli* publicly-available genomes based on nucleotide sequence. Maximum likelihood phylogeny of CA *S. sonnei*, *E.coli*, and publicly-available *S. sonnei* genomes based on genome-wide hqSNPs. Background color: Red- *S.sonnei* from California; Green- other *S. sonnei* from JGI IMG and NCBI databases; Blue- *E.coli* strains from JGI IMG database. Bootstrap threshold 50%.

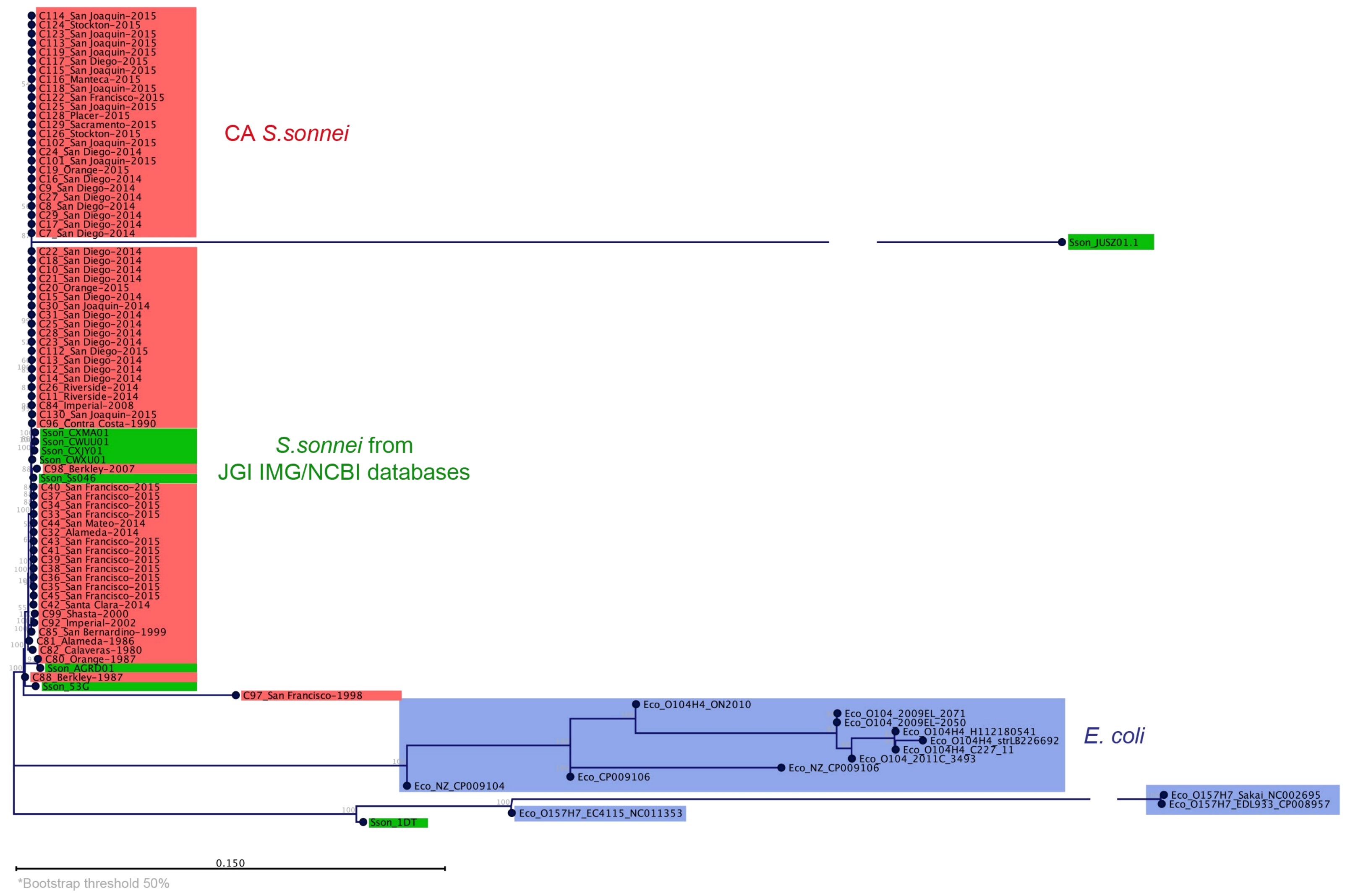

Supplement: Figure S2 [file sph006162211sf3.pdf]
