# Supplementary material for: Recent Outbreaks of Shigellosis in California Caused by Two Distinct Populations of Shigella sonnei with either Increased Virulence or Fluoroquinolone Resistance
Source: mSphere. 2016 Dec 21;1(6):e00344-16. doi: 10.1128/mSphere.00344-16 (PMC5177732; doi:10.1128/mSphere.00344-16)
Supplement: Figure S3 [file sph006162211sf4.pdf]

Figure S3. Comparison of COG abundance profiles of representative CA *S. sonnei* with *E. coli* strains and other *Shigella* species from JGI IMG database.
The heat map represents gene count for different COGs.

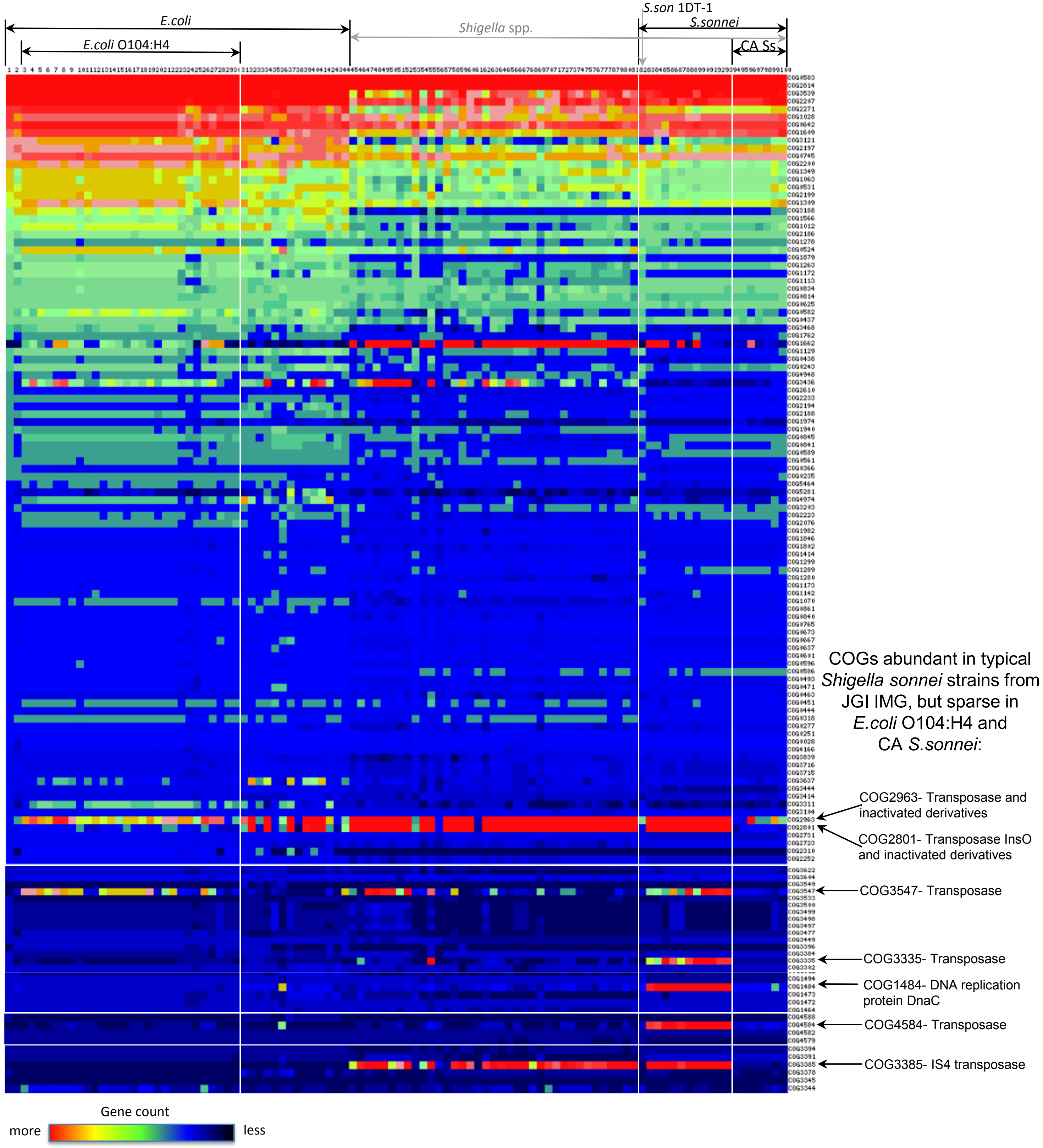

1 - Escherichia coli O103:H25 NIPH-11060424
2 - Escherichia coli O104:H21 CFSAN002236
3 - Escherichia coli O104:H4 04-8351
4 - Escherichia coli O104:H4 09-7901
5 - Escherichia coli O104:H4 11-4404
6 - Escherichia coli O104:H4 11-4632 C3
7 - Escherichia coli O104:H4 2009EL-2050
8 - Escherichia coli O104:H4 2009EL-2071
9 - Escherichia coli O104:H4 2011C-3493
10 - Escherichia coli O104:H4 C227-11
11 - Escherichia coli O104:H4 E112/10
12 - Escherichia coli O104:H4 E92/11
13 - Escherichia coli O104:H4 Ec11-4986
14 - Escherichia coli O104:H4 Ec11-4988
15 - Escherichia coli O104:H4 Ec11-5603
16 - Escherichia coli O104:H4 Ec11-5604
17 - Escherichia coli O104:H4 Ec11-9450
18 - Escherichia coli O104:H4 Ec11-9941
19 - Escherichia coli O104:H4 Ec11-9990
20 - Escherichia coli O104:H4 GOS1
21 - Escherichia coli O104:H4 H112180280
22 - Escherichia coli O104:H4 H112180282
23 - Escherichia coli O104:H4 H112180283
24 - Escherichia coli O104:H4 H112180540
25 - Escherichia coli O104:H4 H112180541
26 - Escherichia coli O104:H4 HUSEC41
27 - Escherichia coli O104:H4 LB226692
28 - Escherichia coli O104:H4 ON2010
29 - Escherichia coli O104:H4 ON2011
30 - Escherichia coli O104:H4 TY-2482
31 - Escherichia coli O111 CVM9455
32 - Escherichia coli O111:H- JB1-95
33 - Escherichia coli O111:H8 CVM9570
34 - Escherichia coli O121:H19 5.0959
35 - Escherichia coli O127:H6 E2348/69 subCVDNalr
36 - Escherichia coli O139:H28 F11 (ETEC)
37 - Escherichia coli O145:H28 RM13514
38 - Escherichia coli O157:H- 493-89
39 - Escherichia coli O157:H7 1044
40 - Escherichia coli O157:H7 EDL933 (EHEC)
41 - Escherichia coli O157:H7 Sakai (EHEC)
42 - Escherichia coli O26:H11 CVM10026
43 - Escherichia coli O55:H7 LSU-61
44 - Escherichia coli O91:H21 96.0497
45 - Shigella boydii 3594-74
46 - Shigella boydii 5216-82
47 - Shigella boydii SB_08-0009
48 - Shigella boydii SB_08-6431
49 - Shigella boydii SB_248-1B
50 - Shigella boydii sv. 4 Sb227
51 - Shigella dysenteriae CDC 74-1112
52 - Shigella dysenteriae S6554
53 - Shigella dysenteriae SD1D
54 - Shigella dysenteriae sv. 1 Sd197
55 - Shigella dysenteriae sv. 4 1012
56 - Shigella dysenteriae WRSd3
57 - Shigella flexneri 12022
58 - Shigella flexneri 1485-80
59 - Shigella flexneri 2850-71
60 - Shigella flexneri 2930-71
61 - Shigella flexneri 4S BJ10610
62 - Shigella flexneri CDC 796-83
63 - Shigella flexneri G1663
64 - Shigella flexneri II:(3)4,7(8)
65 - Shigella flexneri K-1770
66 - Shigella flexneri K-227
67 - Shigella flexneri K-272
68 - Shigella flexneri K-304
69 - Shigella flexneri K-315
70 - Shigella flexneri K-404
71 - Shigella flexneri K-671
72 - Shigella flexneri S5644
73 - Shigella flexneri S5717
74 - Shigella flexneri Shi06HN006
75 - Shigella flexneri sv. 2a 2457T
76 - Shigella flexneri sv. 2a 2457T
77 - Shigella flexneri sv. 2a 301
78 - Shigella flexneri sv. 5 8401
79 - Shigella flexneri sv. 5 8401
80 - Shigella flexneri sv. Fxv 2002017
81 - Shigella flexneri VA-6
82 - Shigella sonnei 1DT-1-
83 - Shigella sonnei 3226-85
84 - Shigella sonnei 3233-85
85 - Shigella sonnei 4822-66
86 - Shigella sonnei 53G
87 - Shigella sonnei Moseley
88 - Shigella sonnei S6513
89 - Shigella sonnei Ss046
90 - Shigella sonnei Ss046
91 - Shigella sonnei SS_08-7761
92 - Shigella sonnei SS_09-1032
93 - Shigella sonnei SS_09-2245
94 - Shigella sonnei STX_CDPH_C113
95 - Shigella sonnei STX_CDPH_C123
96 - Shigella sonnei STX_CDPH_C39
97 - Shigella sonnei STX_CDPH_C7
98 - Shigella sonnei STX_CDPH_C84
99 - Shigella sonnei STX_CDPH_C88
100 - Shigella sonnei STX_CDPH_C97
