# Supplementary material for: Recent Outbreaks of Shigellosis in California Caused by Two Distinct Populations of Shigella sonnei with either Increased Virulence or Fluoroquinolone Resistance
Source: mSphere. 2016 Dec 21;1(6):e00344-16. doi: 10.1128/mSphere.00344-16 (PMC5177732; doi:10.1128/mSphere.00344-16)

Figure S4. Revised pfam phylogeny. Maximum Likelihood clustering of CA representative *S.sonnei* isolates with *E.coli* and other *Shigella* species from IMG JGI database based on pfam profiles (presence/absence).

Background color: Red- representative *S.sonnei* from California; Green- other *S. sonnei* from JGI IMG database; Blue- *E.coli* strains from JGI IMG database.

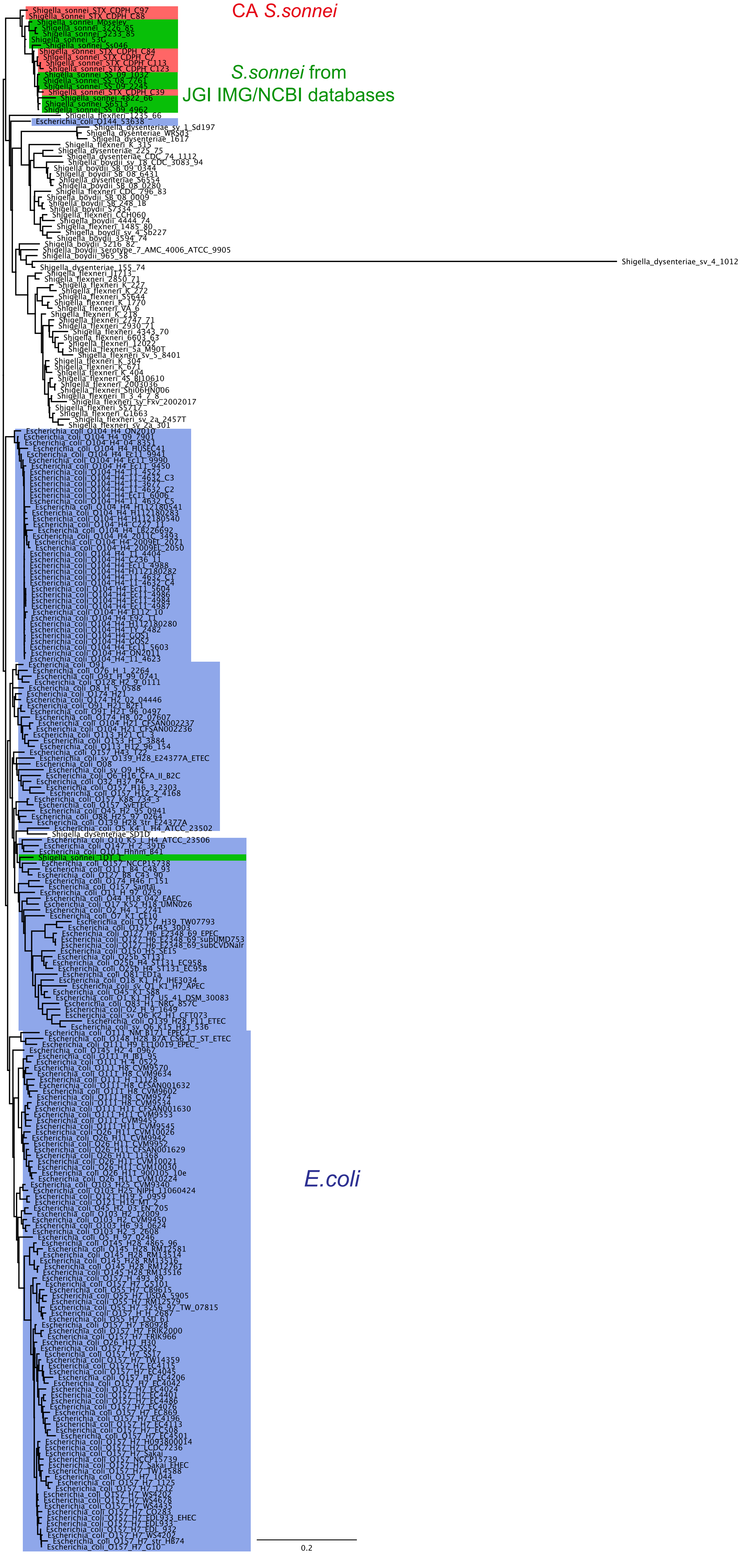

Supplement: Figure S4 [file sph006162211sf5.pdf]
