# Supplementary material for: Recent Outbreaks of Shigellosis in California Caused by Two Distinct Populations of Shigella sonnei with either Increased Virulence or Fluoroquinolone Resistance
Source: mSphere. 2016 Dec 21;1(6):e00344-16. doi: 10.1128/mSphere.00344-16 (PMC5177732; doi:10.1128/mSphere.00344-16)

Figure S5. BLASTn distance tree of top matches to CA STX1-phage nucleotide sequence. Tree building method- Fast Minimum Evolution. Tree is sorted by distance. The numbers over the branches designate branch length. CA *S. sonnei* STX1-phage is highlighted with yellow background color.

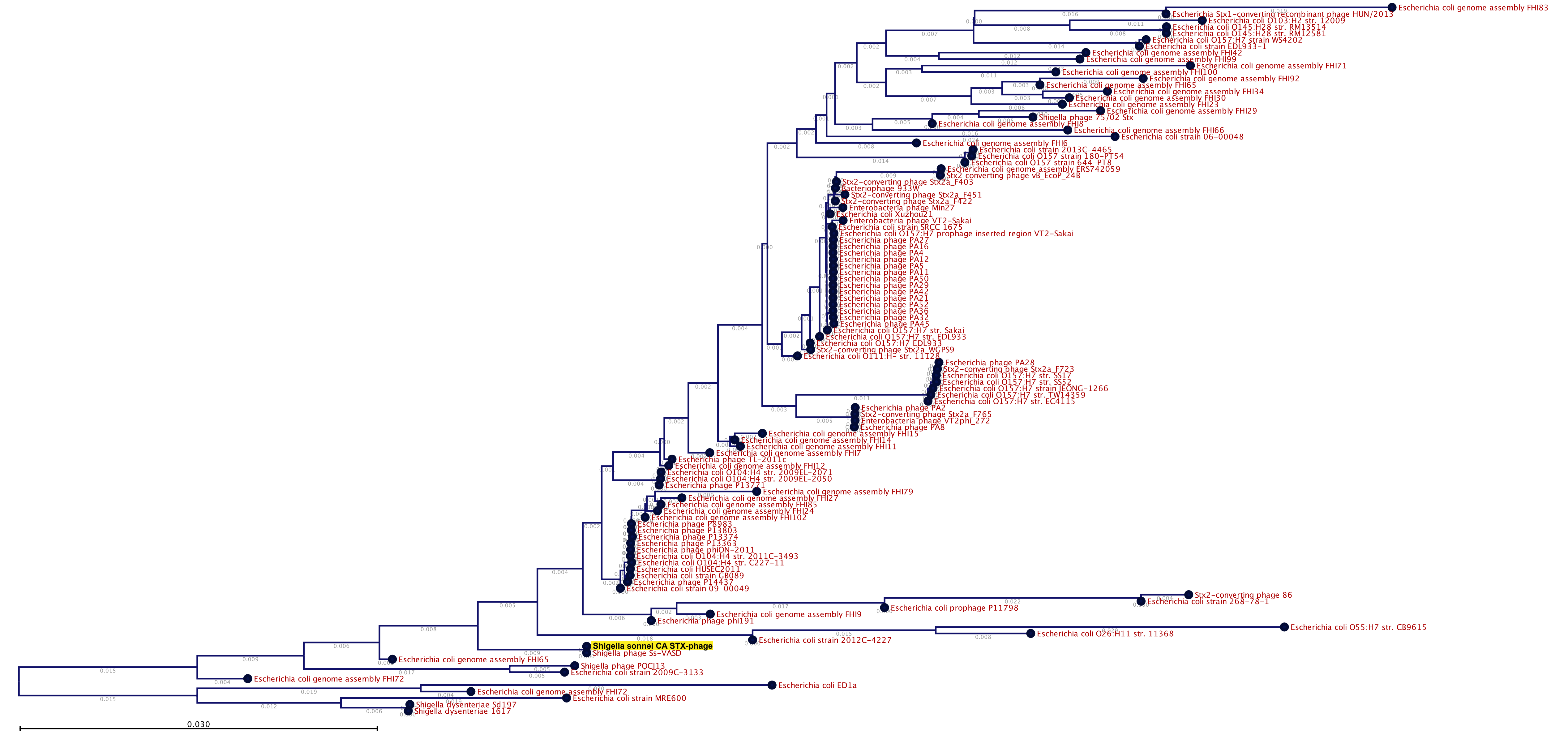

Supplement: Figure S5 [file sph006162211sf6.pdf]
