# Supplementary material for: Recent Outbreaks of Shigellosis in California Caused by Two Distinct Populations of Shigella sonnei with either Increased Virulence or Fluoroquinolone Resistance
Source: mSphere. 2016 Dec 21;1(6):e00344-16. doi: 10.1128/mSphere.00344-16 (PMC5177732; doi:10.1128/mSphere.00344-16)

Figure S6. Phylogeny of the CA STX1-phage. **A.** Neighbor-Joining phylogeny based on amino acid sequence of Integrase protein. **B.** Maximum Likelihood phylogeny based on full nucleotide sequence of the phage. CA STX1-phage is highlighted with yellow background color. The numbers above the branches designate the bootstrap values. Isolates associated with European *E. coli* O104:H4 outbreak of 2011 are highlighted with red label color. The color of the node corresponds to the type of Shiga-toxin.

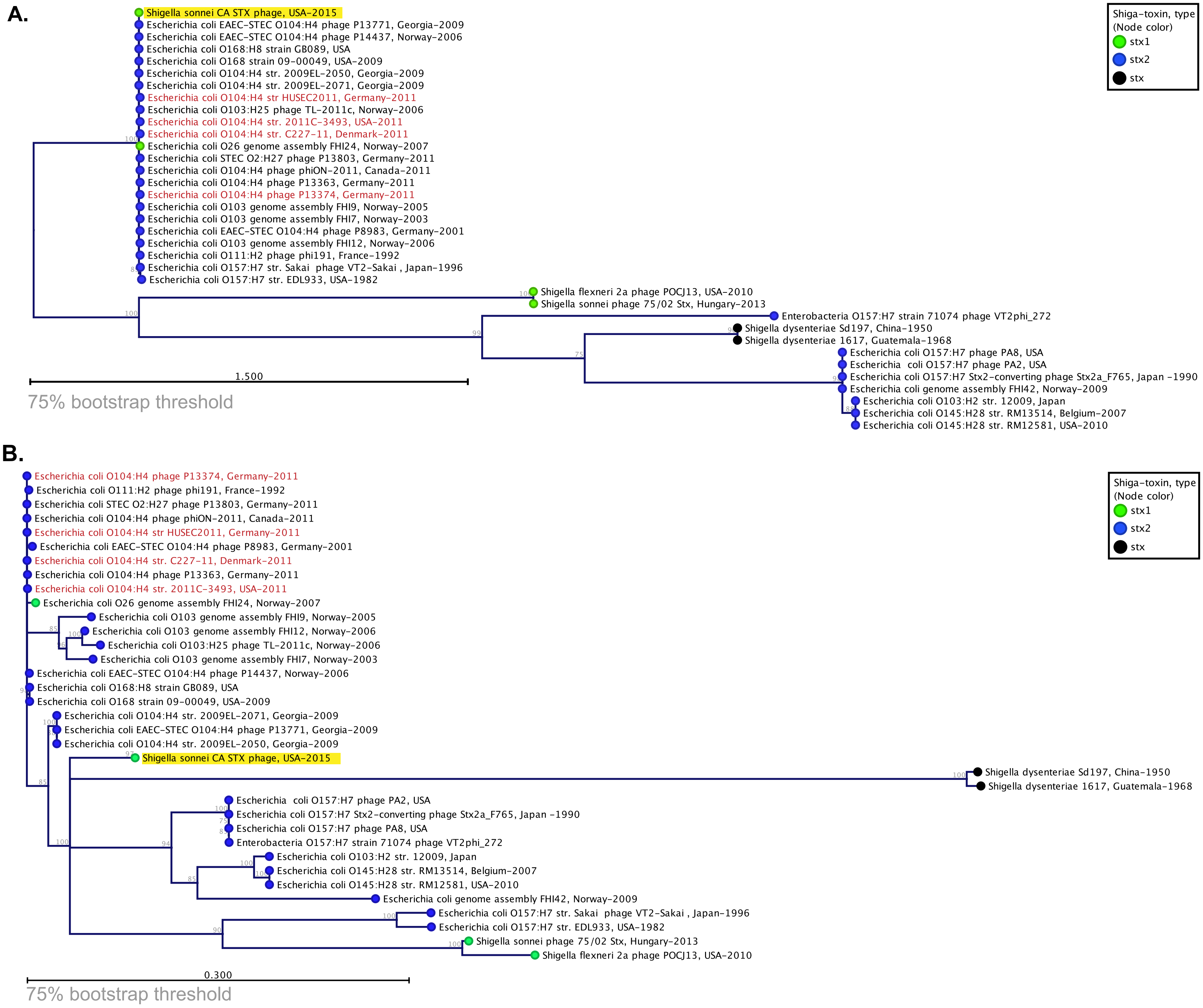

Supplement: Figure S6 [file sph006162211sf7.pdf]
