# Supplementary material for: Recent Outbreaks of Shigellosis in California Caused by Two Distinct Populations of Shigella sonnei with either Increased Virulence or Fluoroquinolone Resistance
Source: mSphere. 2016 Dec 21;1(6):e00344-16. doi: 10.1128/mSphere.00344-16 (PMC5177732; doi:10.1128/mSphere.00344-16)

Figure S7. Cryptic STX-converting prophages found in CA *S.sonnei* genomes.

C7 Region 10- Intact Stx2-converting phage 1717 (NC_011357):

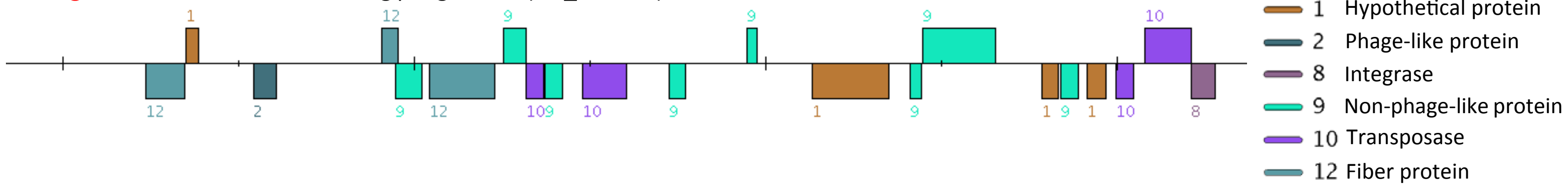

C113 Region 14- Incomplete Stx2-converting phage 86 (NC_008464):

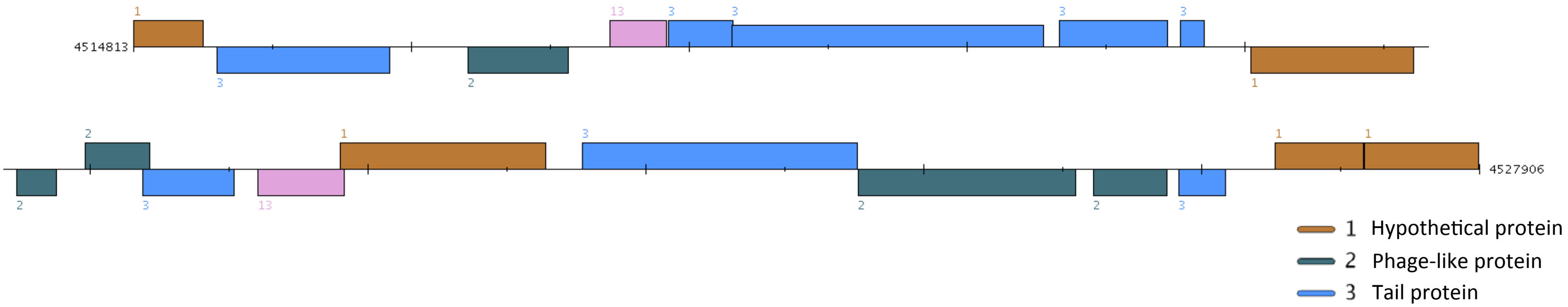

C16-Region 15- questionable Stx2-converting phage I (NC_003525):

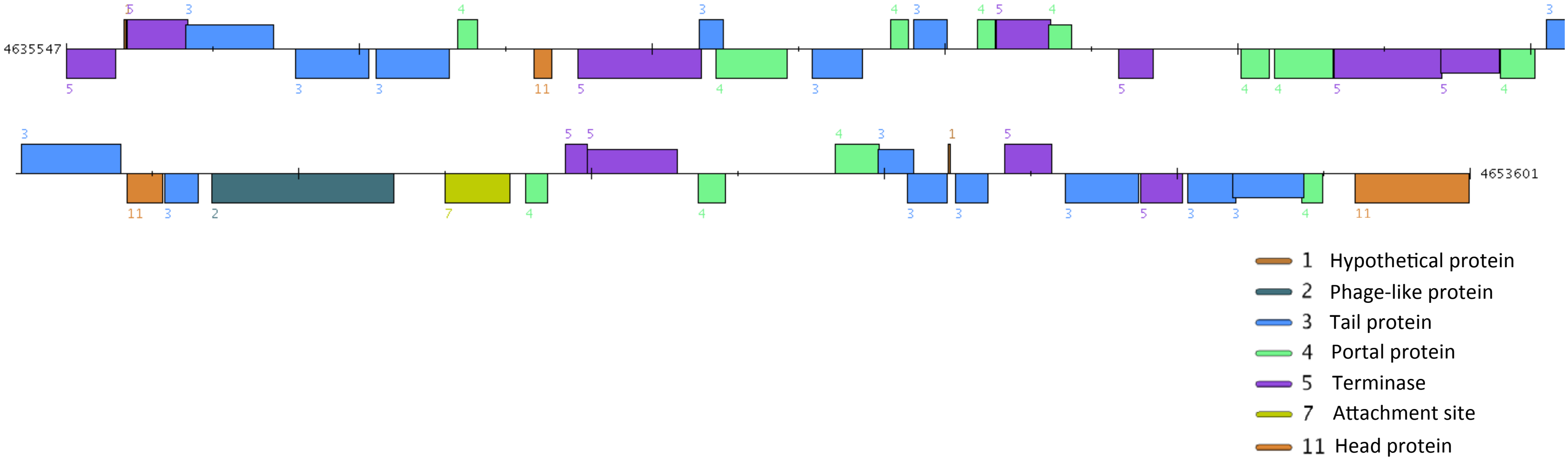

Supplement: Figure S7 [file sph006162211sf8.pdf]
