# Supplementary material for: Recent Outbreaks of Shigellosis in California Caused by Two Distinct Populations of Shigella sonnei with either Increased Virulence or Fluoroquinolone Resistance
Source: mSphere. 2016 Dec 21;1(6):e00344-16. doi: 10.1128/mSphere.00344-16 (PMC5177732; doi:10.1128/mSphere.00344-16)

Figure S8. Plasmids and other mobile elements encoding antibiotic resistance in CA *S. sonnei*.

Abbreviation: hp- hypothetical protein.

A. Organization of *bla*TEM-1- encoding IncB/O/K/Z conjugative plasmid from modern SFr population isolates.

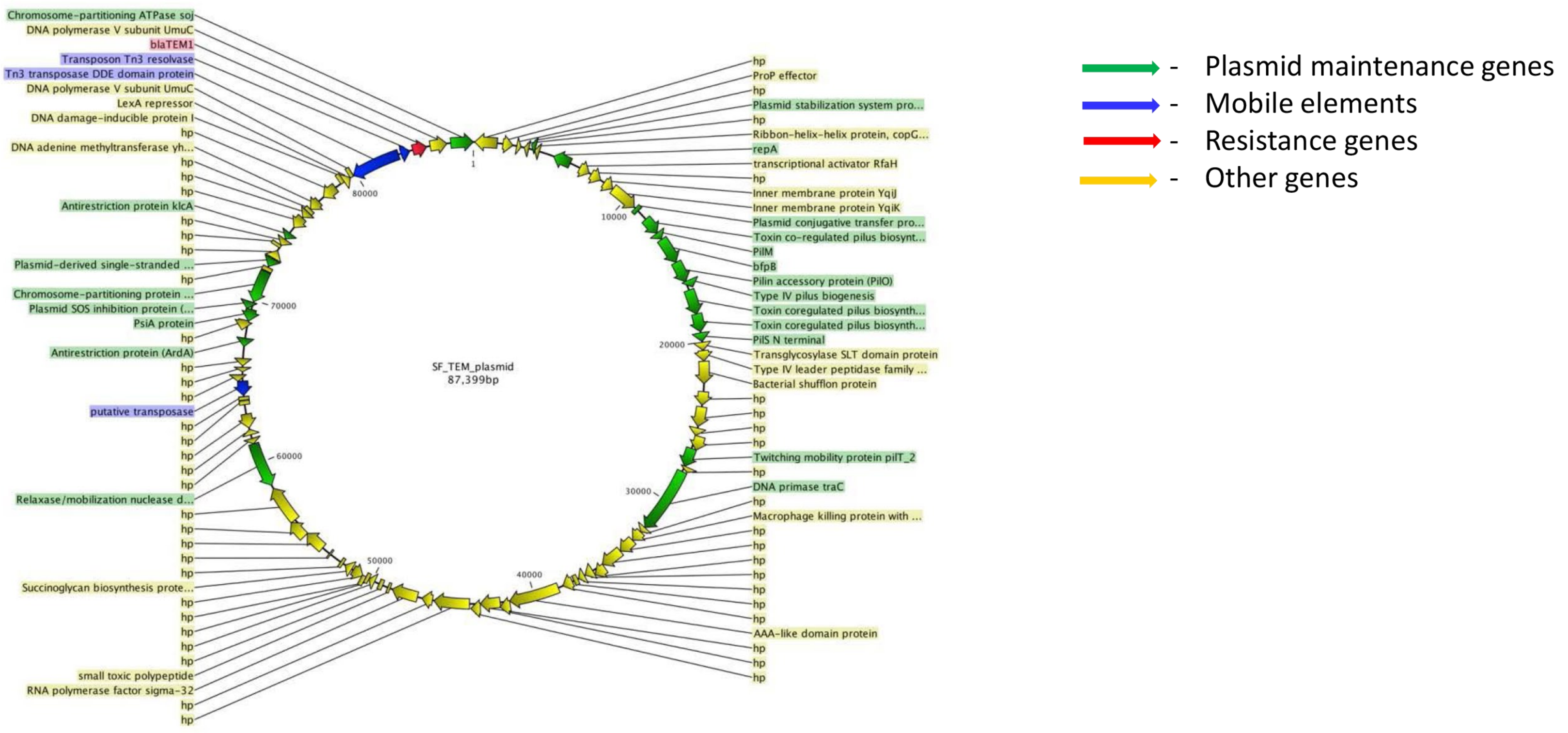

Different integration sites of Tn3::*bla*TEM-1 on IncB/O/K/Z plasmids found in recent SFr isolates and in historical *S. sonnei*:

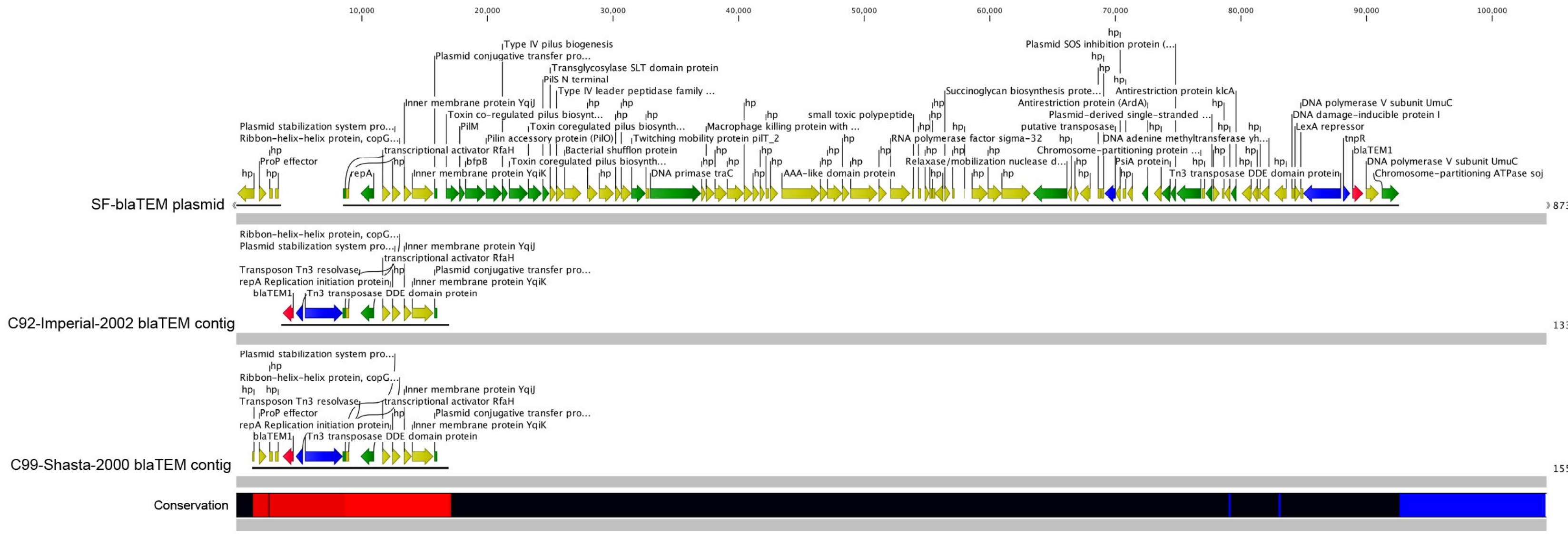

B. A representative genetic surrounding of *bla*TEM-1 gene on a putative IncI1 conjugative plasmid from a modern SJo *S. sonnei* isolate.

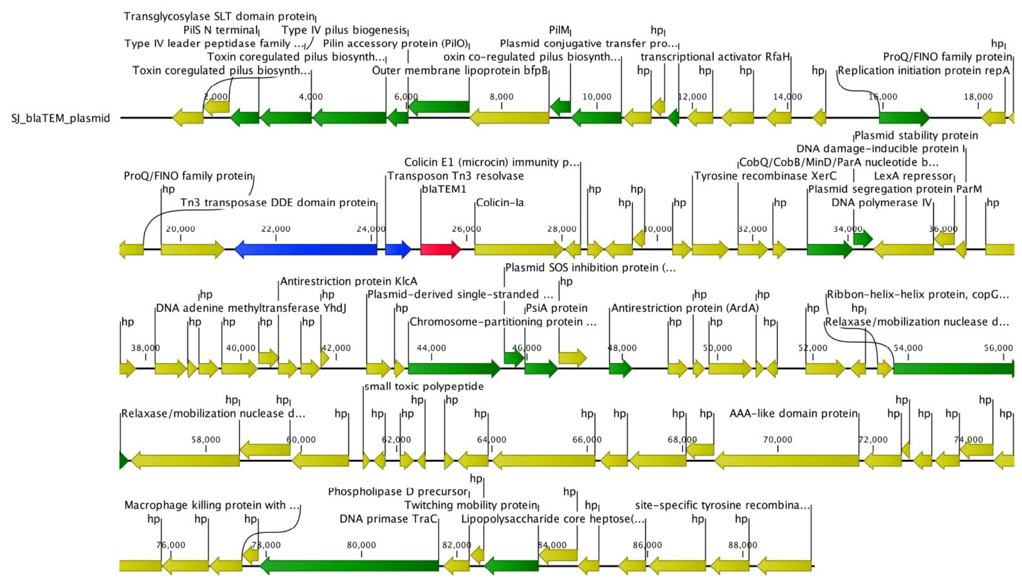

C. Representative organization of *sul2-strA-strB-tetA* resistance genes cluster in SDi/SJo and historical CA *S. sonnei* isolates.

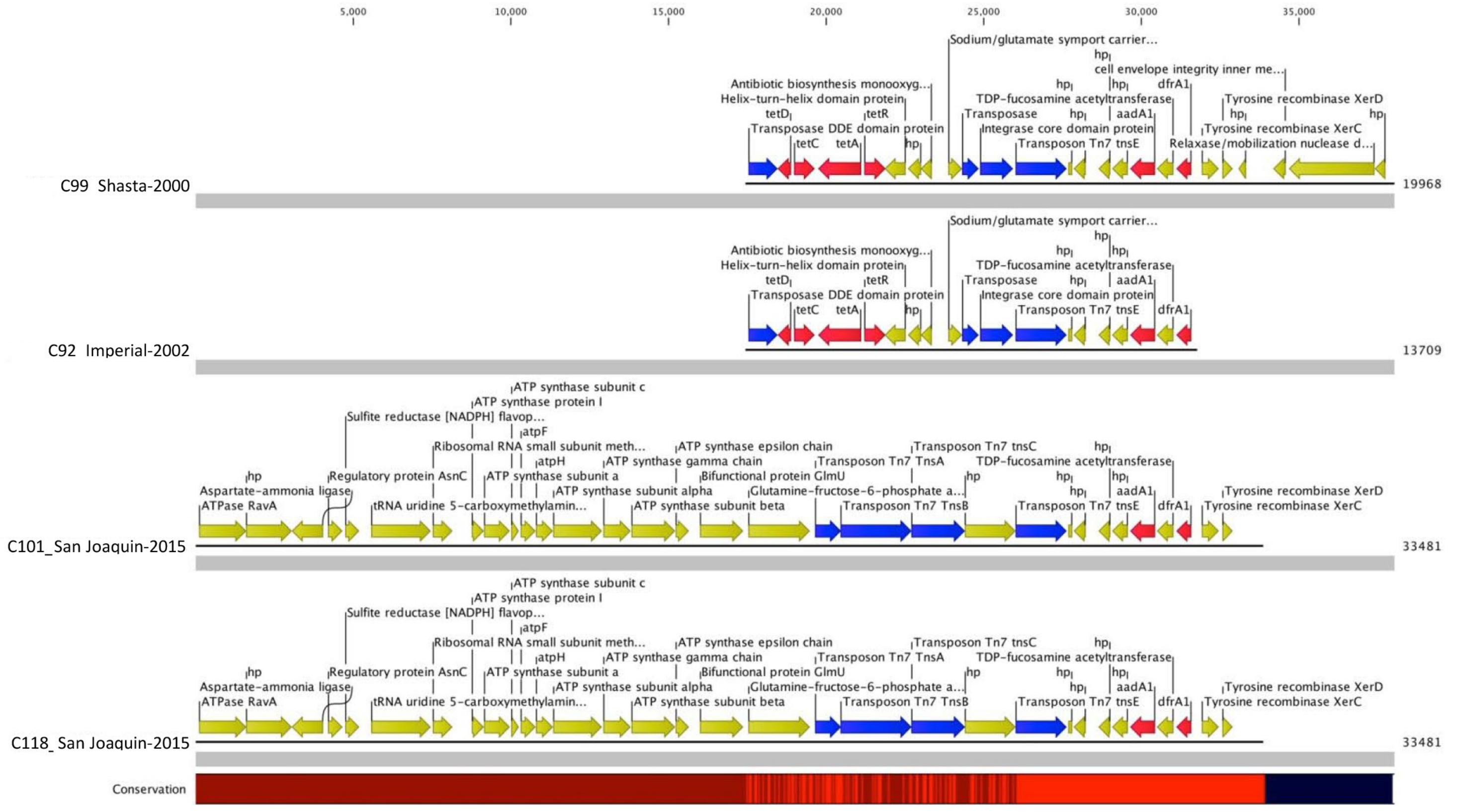

D. The genetic surrounding of macrolide resistance gene in historical *S. sonnei* isolate C80 (Orange County, 1987).

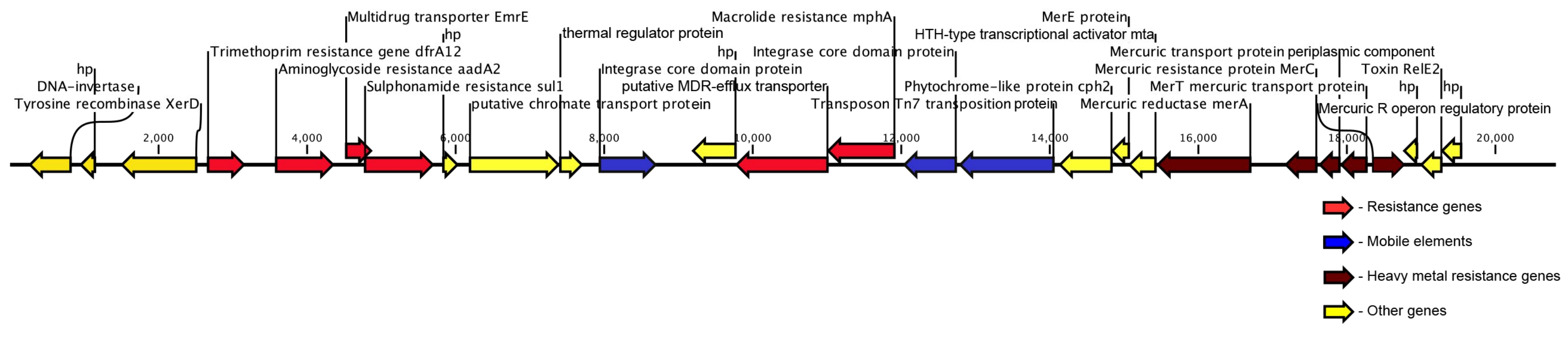

Supplement: Figure S8 [file sph006162211sf9.pdf]
