# Supplementary material for: Recent Outbreaks of Shigellosis in California Caused by Two Distinct Populations of Shigella sonnei with either Increased Virulence or Fluoroquinolone Resistance
Source: mSphere. 2016 Dec 21;1(6):e00344-16. doi: 10.1128/mSphere.00344-16 (PMC5177732; doi:10.1128/mSphere.00344-16)
Supplement: Text S1 [file sph006162211s10.pdf]

Supplementary File 1. Detailed description of high-quality SNP (hqSNP)-based phylogenetic analysis.

For genome-wide SNP identification, trimmed paired-end reads were mapped to the reference genome of *S. sonnei* Ss046 (NC_007384.1) with masking of the mobile and phage elements using CLCbio Genomic Workbench 8.0.2 (Qiagen, Aarhus, Denmark). The mobile and phage elements for masking were predicted using prokka v1.1 [1]and Phage Search Tool (PHAST) [2]. SNPs were called in coding and non-coding genome areas using SAMtools mpileup (v.1.2; [3]) and converted into VCF matrix using bcftools (v0.1.19; http://samtools.github.io/bcftools/). Variants were parsed using vcftools (v.0.1.12b; [4]) to include only high-quality single nucleotide polymorphisms(hqSNPs), which were defined as SNPs with coverage ≥5x, minimum quality > 200, minimum genotype quality (GQ) 10 (--minDP 5; --minQ 200; --minGQ 10; --remove-indels), with InDels and the heterozygote calls excluded. A phylogenetic tree was generated using CLCbio Genomic Workbench 8.0.2 (Qiagen, Aarhus, Denmark) with maximum likelihood phylogeny (under the Jukes-Cantor or General Time Reversible nucleotide substitution models, as specified below; with bootstrapping) based on hqSNPs. Following strain selection and commands were used to investigate California *S. sonnei* phylogeny and global *S. sonnei* phylogeny:

California *S.sonnei* high-quality SNP-based phylogeny

For local California *S. sonnei* phylogeny the whole genome sequences of 68 recent and historical isolates (with at least 30x coverage) were used for phylogenetic inference (Table S1). Following commands and parameters were used to call and filter high-quality SNPs:

```
samtools sort Sample_1_mapped_reads.bam Sample_1_mapped_reads.sorted
```

```
samtools index Sample_1_mapped_reads.sorted.bam

samtoolsfaidx ./NZ_CP010555_PaerFRD1.fa

vcfutils.plsplitchr -l 201062 ./SsonSs046_NC_007384_Transposon.fa.fai
| xargs -I {} -n 1 -P 24 sh -c "samtools mpileup -f
./SsonSs046_NC_007384_Transposon.fa -r '{}' -D -g *.sorted.bam |
bcftools view - >tmp.{}.vcf"

vcf-concat tmp.*.vcf | vcf-sort > res.vcf

bcftools call -c -v res.vcf > res_variants.vcf

vcftools --vcf res_variants.vcf --minGQ 10 --minDP 5 --minQ 200 --
remove-indels --recode --out Shigella_HQ_SNPonly

sed '/\.\/\./d' ./Shigella_HQ_SNPonly.recode.vcf >
Shigella_HQ_SNPonly_sed.recode.vcf

grep -v "0/1" Shigella_HQ_SNPonly_sed.recode.vcf > Shigella_
HQ_SNPonly_nohet.recode.vcf
```

SVAMP software [5] was used to convert final vcf file into fasta alignment (gap-free, pseudo-whole genome sequences).

Fasta alignment of 2,893 SNPs was then used to generate Maximum likelihood tree under the Jukes-Cantor nucleotide substitution model; with bootstrapping.

Global *S.sonnei* high-quality SNP-based phylogeny

To compare local *S.sonnei* populations to international strains, in addition to California isolates the representative sequences of *S. sonnei* from the studies by Holt et al., 2012 [6], Chung The et al, 2015 [7], Chung The et al, 2016 [8] were included into the analysis. The list of 188 analyzed global isolates can be found in Dataset S1. Only the sequences of global strains which yielded the depth of coverage > 10x were included. Genomes of *E.coli* EDL933 (NZ_CP008957.1) and

E.coli Sakai (NC_002695.1) strains were also included into the global phylogenetic analysis, but corresponding nodes were hidden from the final tree due to the space limitation.

All used command were the same as mentioned above for California *S.sonnei* phylogeny, for the exception of following command, which was executed with more strict SNP filtering parameters:

```
vcftools --vcf res_variants_Global.vcf --minGQ 10 --minDP 5 --min-meanDP 30 --minQ 200 --remove-indels --recode --out ShigellaGlobal_HQ_SNPonly
```

The fasta alignment generated as above contained 29,041 SNPs. The Maximum likelihood tree was built under the General Time Reversible nucleotide substitution model; with bootstrapping.

References:

1. Seemann T: Prokka: rapid prokaryotic genome annotation. *Bioinformatics* 2014, 30(14):2068-2069.
2. Zhou Y, Liang Y, Lynch KH, Dennis JJ, Wishart DS: PHAST: a fast phage search tool. *Nucleic acids research* 2011, 39(Web Server issue):W347-352.
3. Li H, Handsaker B, Wysoker A, Fennell T, Ruan J, Homer N, Marth G, Abecasis G, Durbin R, Genome Project Data Processing S: The Sequence Alignment/Map format and SAMtools. *Bioinformatics* 2009, 25(16):2078-2079.
4. Danecek P, Auton A, Abecasis G, Albers CA, Banks E, DePristo MA, Handsaker RE, Lunter G, Marth GT, Sherry ST *et al*: The variant call format and VCFtools. *Bioinformatics* 2011, 27(15):2156-2158.
5. Naeem R, Hidayah L, Preston MD, Clark TG, Pain A: SVAMP: sequence variation analysis, maps and phylogeny. *Bioinformatics* 2014, 30(15):2227-2229.
6. Holt KE, Baker S, Weill FX, Holmes EC, Kitchen A, Yu J, Sangal V, Brown DJ, Coia JE, Kim DW *et al*: Shigella sonnei genome sequencing and phylogenetic analysis indicate recent global dissemination from Europe. *Nature genetics* 2012, 44(9):1056-1059.
7. Chung The H, Rabaa MA, Pham Thanh D, Ruekit S, Wangchuk S, Dorji T, Pem Tshering K, Nguyen Thi Nguyen T, Voong Vinh P, Ha Thanh T *et al*: Introduction and establishment of fluoroquinolone-resistant Shigella sonnei into Bhutan. *Microbial Genomics* 2015.
8. Chung The H, Rabaa MA, Pham Thanh D, De Lappe N, Cormican M, Valcanis M, Howden BP, Wangchuk S, Bodhidatta L, Mason CJ *et al*: South Asia as a Reservoir for the Global Spread of Ciprofloxacin-Resistant Shigella sonnei: A Cross-Sectional Study. *PLoS medicine* 2016, 13(8):e1002055.
